# A comparative approach to elucidate chloroplast genome replication

**DOI:** 10.1186/1471-2164-10-237

**Published:** 2009-05-20

**Authors:** Neeraja M Krishnan, Basuthkar J Rao

**Affiliations:** 1 B-202, Department of Biological Sciences, Tata Institute of Fundamental Research, 1 Homi Bhabha road, Colaba, Mumbai 400 005, India; 2 Current address: Molecular Reproduction, Development and Genetics, Indian Institute of Science, Bangalore 560 012, India

## Abstract

**Background:**

Electron microscopy analyses of replicating chloroplast molecules earlier predicted bidirectional Cairns replication as the prevalent mechanism, perhaps followed by rounds of a rolling circle mechanism. This standard model is being challenged by the recent proposition of homologous recombination-mediated replication in chloroplasts.

**Results:**

We address this issue in our current study by analyzing nucleotide composition in genome regions between known replication origins, with an aim to reveal any adenine to guanine deamination gradients. These gradual linear gradients typically result from the accumulation of deaminations over the time spent single-stranded by one of the strands of the circular molecule during replication and can, therefore, be used to model the course of replication. Our linear regression analyses on the nucleotide compositions of the non-coding regions and the synonymous third codon position of coding regions, between pairs of replication origins, reveal the existence of significant adenine to guanine deamination gradients in portions overlapping the *S*mall *S*ingle *C*opy (SSC) and the *L*arge *S*ingle *C*opy (LSC) regions between inverted repeats. These gradients increase bi-directionally from the center of each region towards the respective ends, suggesting that both the strands were left single-stranded during replication.

**Conclusion:**

Single-stranded regions of the genome and gradients in time that these regions are left single-stranded, as revealed by our nucleotide composition analyses, appear to converge with the original bi-directional dual displacement loop model and restore evidence for its existence as the primary mechanism. Other proposed faster modes such as homologous recombination and rolling circle initiation could exist in addition to this primary mechanism to facilitate homoplasmy among the intra-cellular chloroplast population

## Background

The chloroplast is a vital organelle, responsible for photosynthetic metabolism in plants. Their replication is crucial in ensuring the cellular maintenance and working within plants [1]. Understanding the mechanisms underlying the process of replication can yield important insights that can be used towards plastid engineering and transformation [2], an area in the growing discipline of plant biotechnology. It is, therefore, imperative to delve into and develop our understanding of replication in chloroplast genomes. In the early 1970s, electron microscopy analyses of replicating chloroplast intermediates from pea and corn drew a model of replication [3]. This model was based on two displacement loops (D-loops) separated by some distance on the genome, where the displacement of the two D-loops occur on opposite strands of the parental DNA molecule and subsequently, move towards each other. As a result of this mechanism, half of each displaced parental strand (from either origin until the center of two origins) is left single-stranded on both sides of the pair of inverted repeats. This discovery of Cairn's replication mechanism [4] in pea and corn chloroplast genomes was followed by a series of studies independently confirming this model for various plant species (*Euglena gracilis *[5]; single D-loop]; *Nicotiana tabacum *[6]; *Chlamydomonas reinhardtii *[7]; *Oenothera *[8]; *Zea mays *[9]).

The rolling circle mechanism could be initiated after one round of Cairns type of replication, so as to generate multiple copies of the chloroplast genome even though replication is initiated only once (pea and corn, [10]). Electron microscopy analyses of certain *in vitro *tobacco chloroplast replication intermediates also revealed Y-arc patterns, indicative of rolling circle replication[6].

Bendich and colleagues recently countered the entire proposition of bi-directional replication mediated by two D-loops (see [11] for review) and also the possibility of any rolling circle initiation. The basis for this contrary view is their observation of a large number of molecules in linear or complex branched oligomeric forms [12,13], which were earlier either dismissed as broken circles [14] or physically excluded (by virtue of scientific judgment or ultra-centrifugation methods exercised on the samples prior to electron microscopy analyses). They put forth homologous recombination as the primary mechanism of replication, explaining the generation of all chloroplast replicative intermediates such as oligomeric forms, head to tail concatemers and isomers as well as circular molecules themselves. They further re-iterate the need for a revised view of the standard dual displacement loop model.

We exploit the availability of a large number of chloroplast genomes (>100; see Additional File 1) and adopt a comparative genomics approach in our current manuscript for predicting the chloroplast DNA replication mechanism. It is well-established in animal mitochondrial genomes that replication leaves an imprint on the genome composition, by way of deaminations accumulating during the time spent single-stranded by the parental heavy strand. The adenine to guanine (A → G) deaminations accumulate linearly over the time spent single-stranded during replication while, cytosine to thymine deaminations exhibit a complex, asymptotic response [15,16]. The A → G deamination response is simpler to detect using a linear regression model. One could, therefore, explore the presence of A → G gradients in chloroplast genome regions between mapped replication origins and infer from the direction of these gradients, the direction in which the DNA is left single-stranded during replication. For instance, an increasing gradient of A → G from point 'X' to point 'Y' of the genome indicates that the replication fork proceeded from Y to X causing Y to become single-stranded before X, thereby exposing Y to greater accumulation of A → G deaminations than X.

The tobacco chloroplast genome is best-documented in terms of replication origins [6]. Annotation of the tobacco genome (*Nicotiana tabacum*: NC_001879) in NCBI reveals four origins on each of the two inverted repeats: A1 (35 nucleotides), A2 (82 nucleotides), and two copies of B (243 nucleotides), one on each strand. Formation of D-loops were observed only in those tobacco clones that contained all the origins, and these replication origins were also found to be the minimal sequences to ensure the completion of replication [6]. Replication origins are not annotated in other complete chloroplast genomes, available in NCBI. We use the tobacco genome as a benchmark, to look for homologues of these origins in other genomes and find several matches using the NCBI pair-wise Blast tool. Linear regression analyses reveal for a majority of plant species, significant symmetric bi-directional A → G deamination gradients in the *S*mall *S*ingle *C*opy (SSC) and *L*arge *S*ingle *C*opy (LSC) regions. The single-strandedness window experienced during homologous recombination is too small to result in such nucleotide composition patterns. Secondly, the single-stranded bubble moves as strand invasion progresses during recombination and does not expand like in the case of conventional replication mechanism, thus preventing accumulation of deaminations. On the other hand, the dual displacement loop model [3] very well explains these symmetric A → G gradient trends, suggesting its pre-dominant existence as the mechanism of replication.

## Methods

### Complete Chloroplast Genomes

We analyzed complete chloroplast genomes belonging to 116 plant species, available in NCBI Genbank  as of September 2008. The names of these plant species, their abbreviations and locus IDs are presented in a table (see Additional File 1).

### Identifying homologues to known replication origin sequences

**Figure 1:**
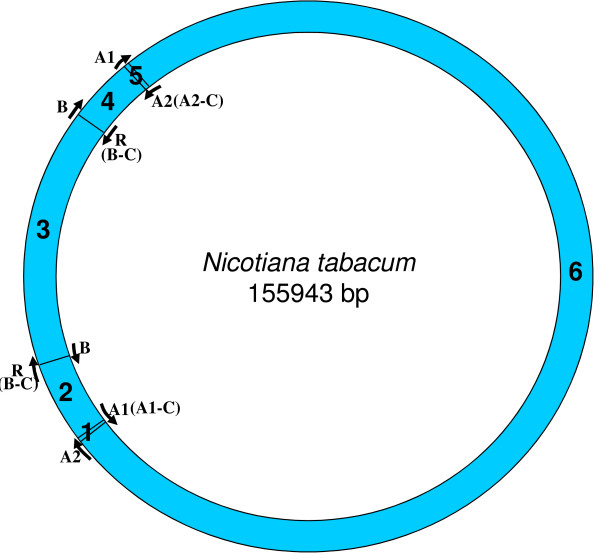
**Locations of replication origins mapped on the tobacco chloroplast genome**. The figure depicts the relative positions of known replication origins (A1, A2, B and R) and their complementary copies (origin type appended with '-C'), on the circular tobacco chloroplast genome, mapped using SimVector 4.22 . The six regions between each pair of replication origins are annotated respectively as 1 (between A2 and A1 [A1-C]), 2 (between A1 [A1-C] and R [B-C]/B), 3 (between R [B-C]/B and B/R [B-C]), 4 (between B/R [B-C] and A1), 5 (between A1 and A2) and 6 (between the A2s on either strand).

**Figure 2:**
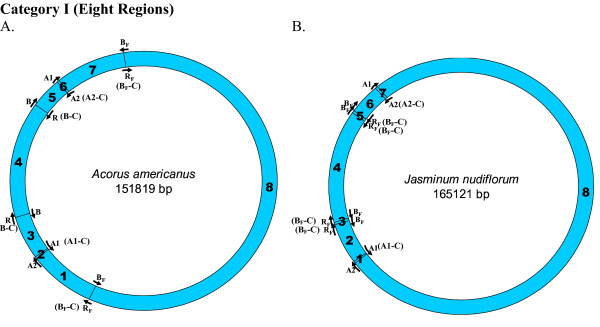
**Mapping of replication origin-like sequence homologues in first category of chloroplast genomes**. The figures show matches of replication-origin sequences mapped on the tobacco chloroplast genome with those in other Viridiplantae chloroplast genome categories, as found using NCBI pair wise Blast tool (E = 0.0001). Chloroplast genomes have been partitioned into seven categories based on the number of homologues found to known replication origins in tobacco. The number of regions between each pair of replication origins, analyzed for the presence of deamination gradients is indicated within parentheses next to each category. Representative genomes from first category are depicted here, with the name of the genome indicated within the circular map. Several categories have more than one representative genome, owing to their overall similar number of matches but differences in the type of matches (see Methods). Genomes of plant species other than those indicated among the representative maps have been tabulated in Table 1, according to their respective fits to match categories.

**Figure 3:**
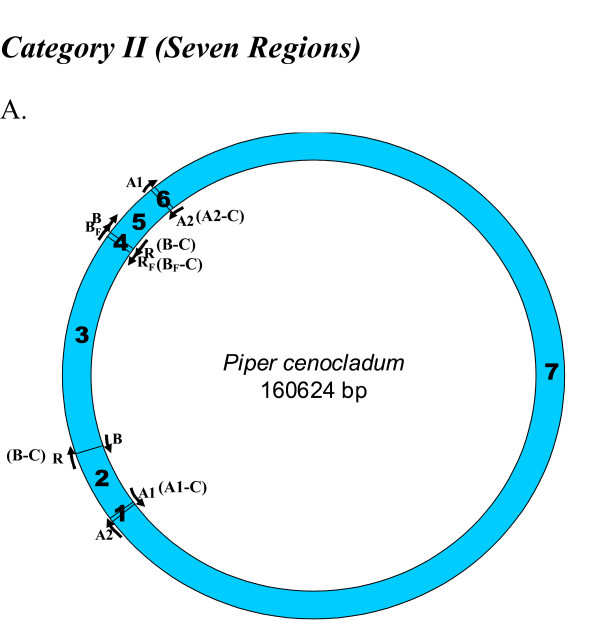
**Mapping of replication origin-like sequence homologues in second category of chloroplast genomes**. Representative genomes from the second category are depicted here. Categorization of chloroplast genomes were performed as described in Figure 2.

**Figure 4:**
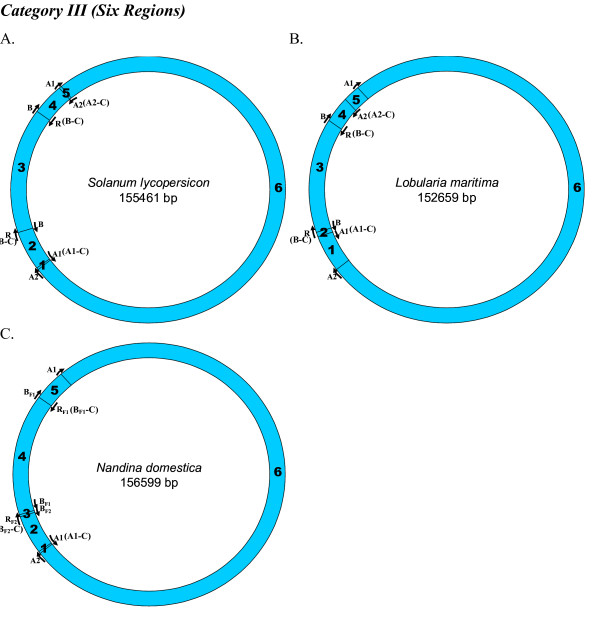
**Mapping of replication origin-like sequence homologues in third category of chloroplast genomes**. Representative genomes from the third category are depicted here. Categorization of chloroplast genomes were performed as described in Figure 2.

**Figure 5:**
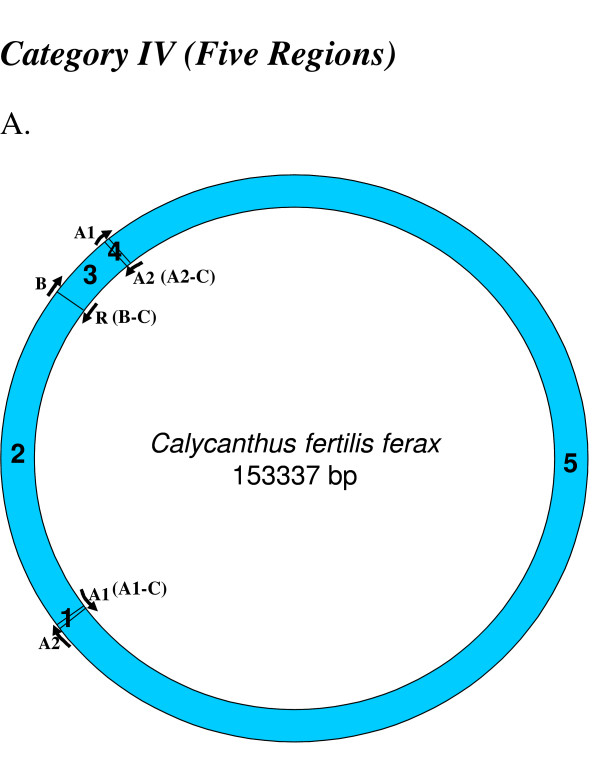
**Mapping of replication origin-like sequence homologues in fourth category of chloroplast genomes**. Representative genomes from the fourth category are depicted here. Categorization of chloroplast genomes were performed as described in Figure 2.

**Figure 6:**
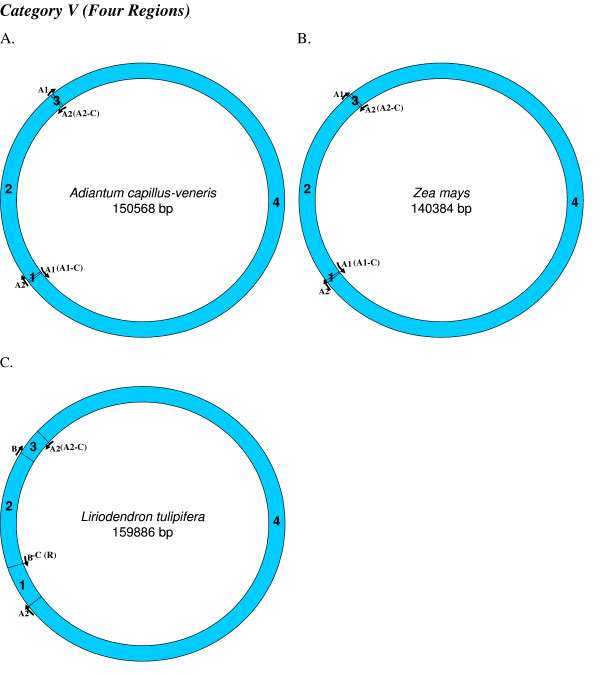
**Mapping of replication origin-like sequence homologues in fifth category of chloroplast genomes**. Representative genomes from the fifth category are depicted here. Categorization of chloroplast genomes were performed as described in Figure 2.

**Figure 7:**
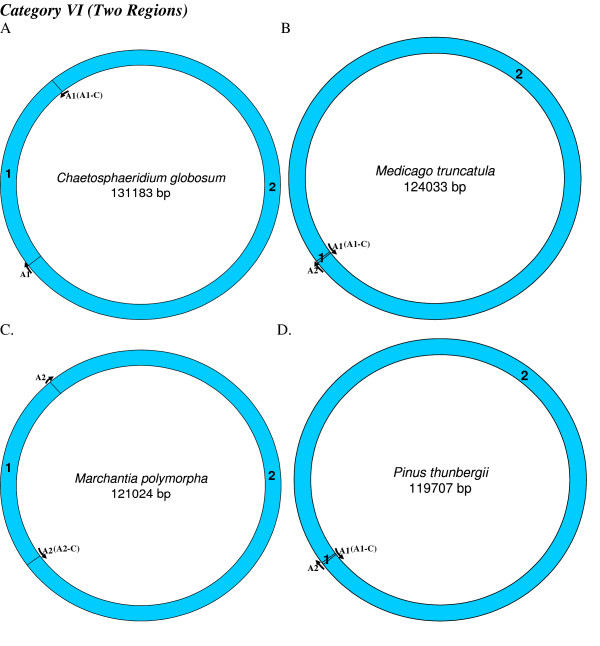
**Mapping of replication origin-like sequence homologues in sixth category of chloroplast genomes**. Representative genomes from the sixth category are depicted here. Categorization of chloroplast genomes were performed as described in Figure 2.

**Figure 8:**
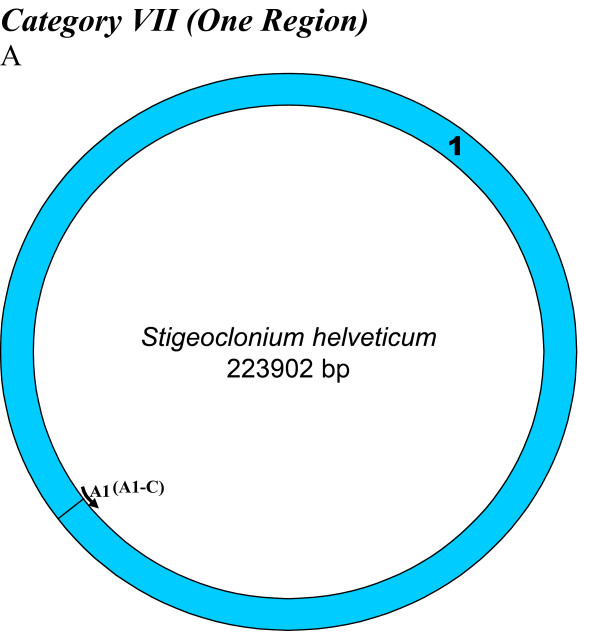
**Mapping of replication origin-like sequence homologues in seventh category of chloroplast genomes**. Representative genomes from the seventh category are depicted here. Categorization of chloroplast genomes were performed as described in Figure 2.

**Figure 9:**
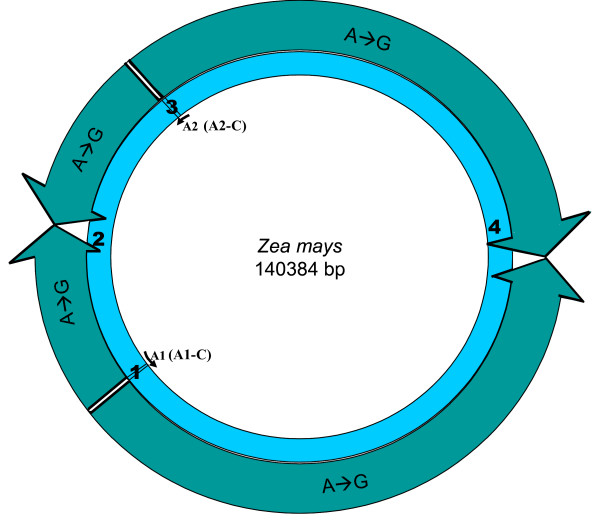
**Minimalist Interpretation of Deamination Gradients in Chloroplast Genomes**. The minimalist model that can be inferred based on our analyses of various regions between replication origins in all chloroplast genomes suggests presence of A → G gradients in the smaller (Type **2**) and larger (Type **4**) regions between A1 and A1-C origin copies and A2 and A2-C origin copies on either inverted repeats, which incidentally overlap with the *S*mall *S*ingle *C*opy (SSC) and *L*arge *S*ingle *C*opy (LSC) regions, respectively. These gradients are bi-directional, increasing from the center of each region towards the origins themselves. The directions of these gradients are shown on the representative genome (*Zea mays)*.

We looked for homologues to the replication origins, A1 (35 bp), A2 (82 bp), B (243 bp) and R (243 bp), known for the tobacco chloroplast genome (positions shown on a circular map; Figure 1), in all other chloroplast genomes. Origins B and R are reverse complementary to each other, and therefore, R is annotated as B-C, from this point onwards in the manuscript. Like-wise, the complementary copies of the other two replication origins, A1 and A2, indicated by counter-clockwise arrows (Figure 1) will be henceforth, referred to as A1-C and A2-C, respectively, in order to distinguish between the two copies. We used the NCBI pairwise Blast tool to perform the sequence homology search and found various numbers of homologues (matches) in various species. In certain instances, we found partial homologues in addition to complete ones, to a given replication origin, within the same genome. These partial matches were included in our analyses and the entire information was used to classify each plant species genome into defined categories. For each genome, we extracted the region of interest between any two replication origin homologues to analyze their nucleotide compositions. The mapping of replication origins, regions of analyses and directions of deamination gradients (Figures 1, 2, 3, 4, 5, 6, 7, 8 and 9) were done using SimVector 4.22 . We performed similar blast analyses for all genomes to a known chloroplast D-Loop sequence in *Chlamydomonas reinhardtii *[17], in order to understand the varying extent of retention of these origin-like sequences in various species, from an evolutionary perspective.

**Table 1:** Categorization of chloroplast genomes based on the numbers of homologues to tobacco chloroplast replication origin sequences

**Category**	**Sub-category**	**Species**
I	[A]	*Aam, Aca*
	
	[B]	*Jnu, Lmi*

II	[A]	*Pce*

III	[A]	*Aco, Agr, Atr, Ath, Abe, Ahi, Bve, Bmi, Cbu, Cpa, Csp, Csi, Car, Cwa, Csa, Cre, Dca, Dne, Dgr, Egl, Fes, Gba, Ghi, Lvi, Les, Mes, Min, Nof, Nsy, Nta, Nto, Nad, Opu, Pal, Pgi, Ptr, Rma, Sbu, Sly, Stu, Sol, Vvi*
	
	[B]	*Lma*
	
	[C]	*Ndo*

IV	[A]	*Cfe, Cfl, Cex, Del, Iol, Nal, Pap, Poc*

V	[B]	*Acav, Pho*
	
	[C]	*Ast, Aev, Afo, Ami, Bdi, Cde, Cta, Evi, Gab, Gma, Han, Hvu, Hlu, Ipu, Lsa, Lpe, Lja, Oar, Obi, Ogl, Oni, Opa, Osa, Pvu, Shy, Sof, Sbi, Tae, Tca, Wmi, Zma*

	[D]	*Ltu*

VI	[A]	*Chgl, Cvu, Cat, Mvi, Pnu, Sob, Ota, Ppa*

	[B]	*Mpo*

	[C]	*Mtr*

	[D]	*Pko, Pth*

VII	[A]	*Cja, Lte, Spu, She, Zci*

VIII		*Cre, Chvu, Hsi, Nol, Ovi, Pak, Sun, Oca*

Species falling into categories I-VIII and further sub-categories are indicated in this table, according to their abbreviations. Sub-categorization is performed within each category, to accommodate more than one representative genome for a given category (Figures 2, 3, 4, 5, 6, 7 and 8). Category VIII comprises of those genomes which do not bear any homology to any replication origin sequence from the tobacco chloroplast genome.

### Linear Regression Analyses

We wrote PERL scripts to extract sequences for each region between a pair of replication origins and further prune them to only consist of non-coding nucleotides and the synonymous third codon position nucleotide, corresponding to an 'A' or a 'G'. This was essential as we were testing for presence of A → G deamination gradients in each region of interest. These positions have relatively lower selection pressure, and hence are likelier to retain and reflect the deamination trends caused during replication. Site-specific adenine (A) to guanine (G) deaminations were estimated as the ratio: A/(A+G), where the nucleotide 'A' at each position was assigned a binary value of 0 and likewise, 'G; was assigned a value of 1. Linear regressions were performed for each extracted region, where the X axes were the positions of nucleotides on the genome, and Y axes values were the estimated site-specific A/A+G ratios. Significance was determined according to a two-tailed t-test (P < 0.05). Similar regression analyses were also performed after splitting the region of interest mid-way into two regions, for each sub-region.

## Results

### Tobacco Chloroplast Replication Origin Homologues in Other Chloroplast Genomes

We found varying numbers of sequence homologues to tobacco chloroplast replication origins in 95 out of 115 other chloroplast genomes (Table 1 and Figures 2, 3, 4, 5, 6, 7 and 8). The local and genomic positions of these homologues are depicted in a table (see Additional File 2). As evident from this table, partial homologues to certain replication origins exist in some genomes, occasionally in addition to their complete homologues. The entire dataset of 115 complete chloroplast genomes could be divided into eight categories, based on the number of similarity matches (including partial matches). Representative genomes from the first seven categories are depicted in Figures 2, 3, 4, 5, 6, 7 and 8. A category often contains more then one representative genome, further depending on the type of matches. For example, Category VI is comprised of four representative genomes, bearing different pairs of replication origin homologues. Such categorization and sub-categorization for all genomes are further represented in Table 1. Among these, III [A] and V [C] sub-categories are highly populated. The eighth category corresponds to genomes (seven green algae: *Chlamydomonas reinhardtii*, *Chlorella vulgaris*, *Helicosporidium sp ex Simulium jonesii*, *Nephroselmis olivacea*, *Oltmannsiellopsis viridis, Oedogonium cardiacum, Pseudendoclonium akinetum *and a blue spike-moss: *Selaginella uncinata*), which lack similarity to any of the tobacco replication origin sequences.

### Significant deamination gradients in certain regions of the genome

We explored the presence of A → G deamination gradients (linearly increasing or decreasing A/A+G ratios) in genome regions interspersed between replication origin homologues. These regions comprised of non-coding and synonymous third codon positions of coding genes. We consistently find significant negative deamination gradients in the region (A2 → A2-C) containing LSC and tail ends spanning ~15kb each of the two inverted repeats for all species belonging to the first six categories (with the exception of *Medicago truncatula *which bears only one inverted repeat; see Additional File 3). Dividing this region (LSC plus tail ends of inverted repeats) mid-way into the two halves, and analyzing each half separately, revealed the presence of two gradients of greater significance, increasing in one half and more strongly decreasing in the other half. Significant A → G gradients in opposite directions were also observed in each half of the genome portion overlapping the SSC region and ~7kb of the other tail end, respectively, of both the inverted repeats (A1 → A1-C) for all species from these six categories. However, the entire SSC region does not exhibit any deamination trend. This is perhaps due to the balancing effect of equally increasing and decreasing trends in both halves. Significant gradients in similar directions were observed in the complete SSC and LSC regions and also within individual halves of these regions, even after excluding the tail ends of inverted repeats suggesting that the detection of these symmetric gradients is not merely influenced by the nucleotide composition of the inverted repeats themselves (data not shown). Directions of these consensus A → G gradients, are depicted on a circular map of the *Zea mays *chloroplast genome (Figure 9). Such significant gradients were not observed consistently across all species in any other region of the genome (see Additional File 3) for these six categories. The seventh category of genomes bears only one replication origin homologue to tobacco origin sequences, and therefore, such symmetric gradients were not observed consistently in them. It is likelier that this category of species have other putative replication origin homologues, pertaining more to lower plant forms (see Discussion)

### Model of Replication in Chloroplast Genomes

**Figure 10:**
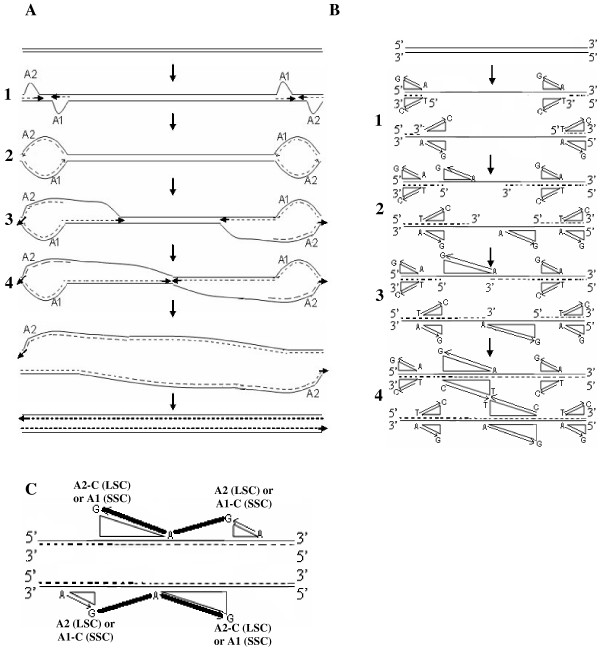
**Generation of single-strandedness and thereby, deamination gradients during the course of chloroplast genome replication**. The cartoon features steps in the course of replication for the region between replication origin pairs on each inverted repeat. The two bubbles represent the displaced parental strands at origins A2 and A1 of each inverted repeat. These bubbles expand towards each other respectively, on each inverted repeat to form a Cairns replication intermediate. Replication forks move in the respective directions to synthesize two complete daughter strands. The parental strands are represented by complete lines, while the daughter strands are represented as dotted lines. The arrows indicate the direction of new strand synthesis. The growing windows of single-strandedness and therefore, A → G deamination gradients are indicated at each step, in parallel (Figures 10A and 10B respectively). The representation in Figure 10C shows the deamination gradients as we observe from analyzing nucleotide compositions of all chloroplast genomes for the region between A1 and A1-C origin copies on each inverted repeat, if we assume it to be overlapping the SSC or the region between A2 and A2-C origin copies, if we assume that it overlaps the LSC. This signature is possible only if replication proceeds as per steps indicated in Figure 10A.

**Figure 11:**
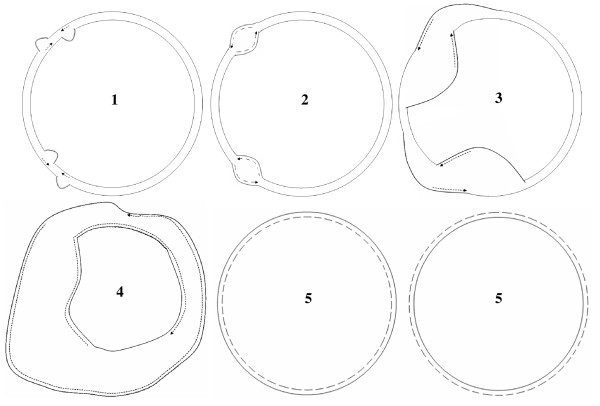
**Mechanism of Replication in the Chloroplast Genome**. This figure shows the steps (1–5) highlighted in Figure 10, on the complete chloroplast genome. The parental strands are represented by complete lines, while the daughter strands are represented as dotted lines. The arrows indicate the direction of new strand synthesis.

We predicted the direction in which the DNA would be left single-stranded during replication based on consensus A → G deamination gradient patterns (Figure 9), in the regions harboring SSC and LSC, and thereby, modeled the mechanism of replication in chloroplast genomes. Inferring from the directions of these gradients, we predict the magnitude of cumulative single-strandedness to be lowest at the ends, i.e. replication origins (A2 and A1) and highest in the middle of SSC and LSC regions. Figures 10A and 10B depict, in parallel, the steps during the proposed process of replication and resultant accumulation of A → G deaminations, respectively, in the SSC region of the genome. The same steps would also hold true for the LSC region of the genome. Our model predicts initiation of replication at the origins, A2 and A1, on both inverted repeats, displacing opposite strands at each origin (Step 1 of Figure 10A). These displacement loops would expand towards each other resulting in Cairn's replicative intermediates (Step 2 of Figure 10A). At this step, if one assumes equal rates of replication forks on both parental strands, one half of the region between A2 and A1 would be left single-stranded for one parental strand, and the other half for the second parental strand, resulting in short gradients in times that the DNA spends single-stranded, from the origins towards the center of the region (Step 1 of Figure 10A). This would establish gradients in deaminations proportional to the gradients in times spent single-stranded by the parental DNA, on both strands (Step 1 of Figure 10B). The proposed Cairn's replicative forks further progress according to this model, each in their respective directions, opposite from one another, generating partial single-strandedness in both strands (Steps 3 and 4 of Figure 10A). As a result, the smaller and larger (SSC and LSC, respectively) regions between the Cairn's intermediates, would be left single-stranded, one half for one parental strand and the other half for the other parental strand (Step 4 of Figure 10A). Gradients in single-strandedness would be established in each half of the SSC and LSC regions, from the location of proposed Cairn's structures towards the center of these regions. Consequently, the A → G deaminations would accumulate proportionally to the predicted gradients in time that the parental DNA is left single-stranded (Steps 2–4 of Figure 10B). Figure 10C depicts the imprint that would be left by a round of chloroplast genome replication as per our model, on the genomic composition: more G's at the ends of SSC and LSC regions and more A's in the middle of these regions, which also matches with our statistical analyses of deaminations in these regions. The model of replication depicted for the linear stretch corresponding to SSC (Figure 10), is expanded to the entire circular genome (Figure 5) and the predicted generation of single-strandedness is shown in a temporal sequence (Steps 1–5; Figure 11). The model derived in our study, using an independent approach based on comparative genomics, surprisingly converges with the dual displacement loop model first put forth by the Kolodner group, based on electron microscopy analyses.

## Discussion

### Deamination trends caused by cumulative single strandedness during replication

Accumulation of deaminations has been attributed to single-strandedness during replication in bacterial [18-21] as well as animal mitochondrial genomes [15,16,22-24]. Both these types of genomes also report strand asymmetry in nucleotide compositions [22,24,25]. A close attempt to studying strand asymmetry in nucleotide composition in chloroplast genomes failed to detect the patterns we observe, because of their approach of studying the entire strand at once [26]. Strand switching asymmetry at the replication origins was however observed in the *Euglena gracilis *chloroplast genome, such that one strand is AC rich and the other is GT rich [27].

**Figure 12:**
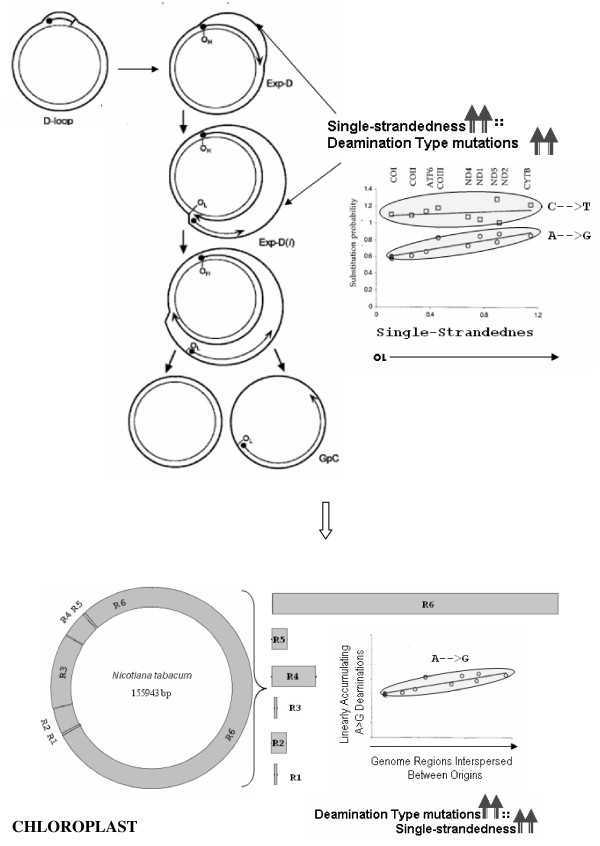
**Hypothesis for inferring the mechanism of replication in chloroplast genomes**. The schematic shows extension of insights developed in animal mitochondrial genome systems, where deamination gradients have been related to single-strandedness during replication, to the plant chloroplast system. The hypothesis developed here is to test for the presence of local deamination gradients in regions spanning between replication origins (1–6), from which the direction in which the DNA is left single-stranded during replication could be inferred, to arrive at a model for the mechanism of chloroplast replication. The left portion of the image in the top rectangle, demonstrating mitochondrial replication is modified from Figure 1 in [60], and the graph on the right side of this image depicting genome-wide C → T and A → G deamination gradients is modified from Figure 4 in [23], respectively, such as to reflect heavy strand notations.

Cytosines to thymines are more rapidly occurring deaminations than adenine to guanine mutations [28,29]. However, the strand biases are caused more prominently by the gradually accumulating adenine to guanine deaminations as shown for animal mitochondrial genomes [24]. Indeed the substitution rates for cytosine to thymine (C → T) deaminations exhibit a complex asymptotic response, where they accumulate very rapidly for shorter times spent single-stranded and saturate above a certain threshold of time spent single-stranded by the genome, perhaps due to a repair mechanism [15,16,24]. The C → T trend is therefore mostly flat after a steep rise, whereas the A → G deaminations exhibit a gradual linear trend [15,16,24]. Consequently, we used the presence of A → G deaminations as an indicator of single-strandedness, according to the assay described in Figure 12.

Chloroplast genomes are also known to undergo heavy RNA-editing, where C → U and U → C mutations occur at the first two codon positions of protein coding genes [30-32] and also certain non-coding regions [33] during transcription. RNA level variation is brought about as a result of such editing to a level that is complementary to DNA level variation [34]. C to U editing is thought to be invoked by deaminations rather than specific nucleotide excision/replacement or trans-glycosylation pathway [35-37]. This suggests the existence of mechanisms to regulate RNA-editing within the chloroplast system, which could also putatively affect C → U (T) deaminations that occur during replicative single-strandedness. This speculation is strengthened by the finding of a chloroplast specific apparatus responsible for editing of *Zea mays *plastid mRNAs [38]. C → T deaminations have also been reported in association with single-strandedness during transcription in *E. coli*, by formation of RNA-DNA hybrids and thereby, C → T mutations accumulating on the non-transcribed strand [39,40]. In order to clearly interpret the nucleotide composition trends as those to have resulted from replication, we rely solely on A → G deamination gradient analyses to infer single-strandedness of the genome during replication.

### Number of Homologues: Genome Size and Evolutionary Trends

**Figure 13:**
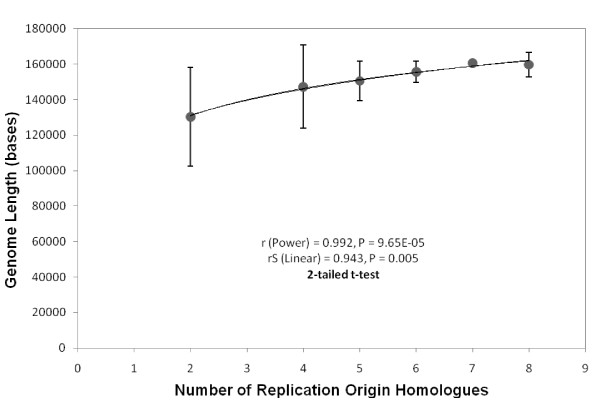
**Relationship between number of homologues to tobacco chloroplast replication origins and length of the genome**. The lengths are averaged across genomes within each category. The data point corresponding to category 1 does not fit in this positive trend, and is therefore excluded from this plot.

The model of replication proposed using the directions of deamination gradients, holds true for the first six categories of genomes (see Additional File 3), where we find consensus and symmetric gradients between replication origin homologues. The total number of homologues to tobacco chloroplast replication origin sequences, including partial ones, are strongly and positively correlated with the size of the genomes (r = 0.992, P = 0.0001 and rS = 0.943, P = 0.005; Figure 13), after excluding the seventh and eighth categories. Changes in genome size along with changes in other co-evolving factors such as cell size, seed mass, stomatal density, photosynthetic rates and specific leaf area [41], leaf strategy and metabolic rates, are thought to play a role in determining ecological [42] and life history strategies of the plant species [43-47].

**Figure 14:**
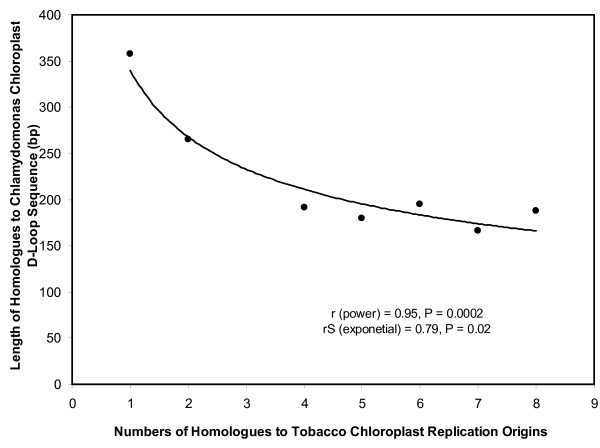
**Relationship between numbers of replication origins homologous to those in tobacco and the extent of homology to a *Chlamydomonas *D-Loop sequence in Viridiplantae chloroplast genomes**. The extents of homology are measured by the size of the region (nucleotides), in Viridiplantae chloroplast genomes that is homologous to a *Chlamydomonas *D-Loop sequence [17].

It is possible that there are other putative replication origins for genomes with one or no homologues to tobacco sequences. We do find the numbers of replication origins homologous to those of tobacco for each genome to be inversely correlated with the extent of homology in these genomes to a known D-loop sequence of *Chlamydomonas reinhardtii *[17], a uni-cellular alga (Figure 14). The extent of homology in this case is quantified as the length of the sequence in various chloroplast genomes that is homologous to the D-Loop sequence of *Chlamydomonas reinhardtii*. This inverse relationship suggests that there could be alternative replication mechanisms for species belonging to category VIII and even VII, perhaps following a similar bi-directional replication mode using origin homologues overlapping those used by algal species more than those of higher plant species.

### Symmetric Gradients in Bacterial Genomes: Similarity in Replication processes

We also find symmetric A → G gradients in opposite directions in regions between replication origins for an *E.coli *Pola 52 plasmid carrying three replication origins (ori-alpha, ori-beta and ori-gamma; see Additional File 4), especially in the larger region between ori-beta and ori-alpha (R3). This plasmid was chosen for analyses as it carries multiple replication origins. The symmetric increasing and decreasing gradients in the larger region between ori-beta and ori-alpha very well fits with *in vivo *evidence for clockwise and counter-clockwise modes of replication from ori-beta and ori-alpha, respectively, known for the highly replicative R-plasmid (R6K) and its derivatives in *E.coli*, carrying a similar arrangement of vegetative replication origins [48].

Ethidium bromide stained fluorescence microscopy images of the nucleoids of *Borrelia burgdorferi *and *Borrelia hermsii *were observed to be different from that of the *E. coli *nucleoid [49,50], and more similar to that observed for chloroplast DNA of maize [12], *Arabidopsis *[51], pea, tobacco and *M. truncatula *[52], especially when the DNA is extracted from young tissue at very early stages of development [52]. These observations provide some evidence for the existence of developmentally regulated homologous recombination in Borrelia genomes. Strand-specific asymmetry has been shown to exist also in genomes of Borrelia species [24]. Since replication mediated by homologous recombination does not generate enough cumulative single-strandedness to result in strand asymmetric nucleotide compositions or gradients, therefore, bi-directional replication using multiple origins could also be possible in Borrelia genomes.

### Mixed Modes of Replication

The relative abundance of oligomeric forms observed for chloroplast DNA stays constant through all stages of leaf development, as found in the case of spinach [53], triggering the possibility of intra-molecular recombination between inverted repeats [54], to generate such multimeric intermediates through a process [55], similar to yeast 2 μm plasmid replication. These plasmids follow Cairn's mechanism of replication after initiation at a single origin. This is trailed by further amplification through intra-molecular recombination between the inverted repeats, after the replication fork passes through one of the inverted repeats [56], such that the two replication forks now chase one another, thereby resembling a double rolling circle, a process also referred to as copy-choice recombination during replication [57].

Homologous recombination cannot explain the generation of nearly symmetric consensus gradients in A → G deaminations in the SSC and LSC regions of the chloroplast genomes as observed by our approach, since the single-strandedness generated during this process is small and non-cumulative. Nevertheless, mixed modes of traditional Cairn's replication via origin firing as well as replication slippages [57] following recombination could indeed occur in chloroplast DNA [55]. The high rate of homologous recombination between multiple circular chloroplast DNA molecules present in close physical proximity inside a single chloroplast can as well bring about efficient homoplasmy [58-60].

### Dynamic Network of Chloroplast Genomes

With advent of approaches that monitor DNA dynamics in living cells, animal mitochondria were found to not exist as autonomous individual organelles but instead form a highly dynamic semi-tubular network. It is possible that the arrangement of ethidium-stained chloroplast DNA as clots and comets with extended fibers as observed by fluorescence microscopy visualization [12] resembles foci formed by ethidium-stained DNA on the dynamic tubular network arrangement of mitochondria in human living cells [61]. Quite analogous to an average of eight genome equivalents found in these highly branched chloroplast DNA structures [12], the number of genomes in human mitochondrial DNA foci also vary from six to ten [61]. In the case of human mitochondria, the model of replication where each genome acts as an individual unit and replicates independently, even while being part of a focus appears as the one most satisfying the observed kinetics. Similarities between structural-functional organizations of these organelles, predicts such independent genome replication model also for chloroplast DNA. A mixed mode of replication could be followed by chloroplast genomes, even in this dynamic network like arrangement.

### Minimalist Model of Chloroplast Replication

The minimalist model of chloroplast replication presented here (Figure 11), excludes ori-B pairs on each inverted repeat and is based on A1 and A2 origins alone. This is because we observe significant symmetric gradients for genome categories V and VI, which lack ori-B homologues. Excluding the ori-B homologues from genomes belonging to the first four categories, we find similar significant symmetric gradients in regions overlapping the SSC and LSC respectively. The presence or absence of ori-B determines *in vitro*, whether the D-loop mode or rolling circle mode of replication is adopted as the predominant mechanism in tobacco chloroplast DNA [6]. It is possible that the ori-B sequences act as accessory units in the strand displacement process, after replication initiation at the A2 and A1 origins. These stretches could also putatively assist intra- or inter-molecular homologous recombination, to result in branched oligomeric structures, as found by Bendich and colleagues. It is nevertheless surprising to note that the minimalist model of replication derived using an independent approach based on comparative genomics, resembles the initial model of dual displacement loop mode of replication, suggesting that it prevails at least during advanced developmental stages.

## Conclusion

Model of chloroplast replication as inferred by analyzing local deamination gradients in regions between replication origins conforms to the bi-directional replication model put forth by the Kolodner group. Homologous recombination could exist as an alternate or additional mechanism.

## Authors' contributions

NMK and BJR conceived the idea of exploring chloroplast genome nucleotide composition. NMK devised the comparative approach to identify consensus nucleotide composition patterns in chloroplast genomes that could result from single-strandedness during replication, and subsequently put forth a model of single-strandedness based on consensus deamination gradient patterns. NMK further shortly extended this approach to bacterial plasmid DNA. NMK and BJR have read and given final approval of the version to be published.

## Supplementary Material

Additional File 1 **List of Complete Chloroplast Genomes**. Shown in this table are the names, abbreviations and locus IDs of 116 species for which complete chloroplast genomes were available in NCBI GenBank as of September 2008. Click here for file

Additional File 2 **Locations of complete and partial sequence homologues to the tobacco chloroplast replication origin sequences**. Begin and end limits are shown in this table for replication origin homologues of each sub-category of genomes, for the replication origin as the query (Q) sequence and the complete chloroplast genome as the subject (S) sequence. Reverse complementary sequence homologues to any of the replication origins (A1, A2, B and R) are indicated as '-C'. Partial homologues to any replication origin sequence are represented as the abbreviation for that origin, superscripted with ^P^. Blast analyses were performed using NCBI's pairwise blast tool . Click here for file

Additional File 3 **t-statistics of the regression slopes between (A/A+G) ratios at each site and their relative positions on the genome, in regions between homologues to tobacco chloroplast replication origin sequences**. The t-statistics of the A → G (i.e. A/(A+G)) deamination trends (i.e. of regression slopes between A/A+G ratios at each site and the relative position of this site on the genome) are depicted for all sub-categories of genomes in this table, for the complete regions between homologues to tobacco chloroplast replication origins (first row for each species appended with '-C'), and after dividing these regions mid-way, for the first half (second row for each species appended with '-I') and second half (third row for each species appended with '-II') respectively for these regions. Significant values (*p *< 0.05, 2-tailed t-test) are emboldened. Click here for file

Additional File 4 **Symmetric A → G deamination gradients in *E. coli *plasmid**. The replication origins (ori-alpha, ori-beta and ori-gamma) are mapped on the circular pOLA52 plasmid of *E. coli *(NC_010378) using SimVector 4.22, and the regions between these origins are annotated as R1, R2 and R3, respectively (see Additional File 4A). The t-statistics of the A → G (A/(A+G)) deamination trends are depicted in the table (see Additional File 4B, for the complete regions R1, R2 and R3 (first row for each species appended with '-C'), and after dividing these regions mid-way, for the first half (second row for each species appended with '-I') and second half (third row for each species appended with '-II') respectively for these regions. Significant values (P < 0.05, 2-tailed t-test) are emboldened. Click here for file
