# Supplementary material for: A comparative approach to elucidate chloroplast genome replication
Source: BMC Genomics. 2009 May 20;10:237. doi: 10.1186/1471-2164-10-237 (PMC2695485; doi:10.1186/1471-2164-10-237)
Supplement: Additional File 1 — List of Complete Chloroplast Genomes. Shown in this table are the names, abbreviations and locus IDs of 116 species for which complete chloroplast genomes were available in NCBI GenBank as of September 2008. [file 1471-2164-10-237-S1.pdf]

Sr. No.	Species	Abbreviations	Locus IDs
1	*Acorus americanus*	*Aam*	NC_010093
2	*Atropa belladonna*	*Abe*	NC_004561
3	*Acorus calamus*	*Aca*	NC_007407
4	*Adiantum capillus-veneris*	*Acav*	NC_004766
5	*Aethionema cordifolium*	*Aco*	NC_009265
6	*Angiopteris evecta*	*Aev*	NC_008829
7	*Anthoceros formosae*	*Afo*	NC_004543
8	*Aethionema grandiflorum*	*Agr*	NC_009266
9	*Arabis hirsuta*	*Ahi*	NC_009268
10	*Aneura mirabilis*	*Ami*	NC_010359
11	*Agrostis stolonifera*	*Ast*	NC_008591
12	*Arabidopsis thaliana*	*Ath*	NC_000932
13	*Amborella trichopoda*	*Atr*	NC_005086
14	*Brachypodium distachion*	*Bdi*	NC_011032
15	*Buxus microphylla*	*Bmi*	NC_009599
16	*Barbarea verna*	*Bve*	NC_009269
17	*Coffea arabica*	*Car*	NC_008535
18	*Chlorokybus atmophyticus*	*Cat*	NC_008822
19	*Capsella bursa-pastoris*	*Cbu*	NC_009270
20	*Ceratophyllum demersum*	*Cde*	NC_009962
21	*Cuscuta exaltata*	*Cex*	NC_009963
22	*Calycanthus fertilis var. ferax*	*Cfe*	AJ428413
23	*Calycanthus floridus var. glaucus*	*Cfl*	NC_004993
24	*Chaetosphaeridium globosum*	*Cgl*	NC_004115
25	*Chlamydomonas reinhardtii*	*Chre*	NC_005353
26	*Chlorella vulgaris*	*Chvu*	NC_001865
27	*Cryptomeria japonica*	*Cja*	NC_010548
28	*Carica papaya*	*Cpa*	NC_010323
29	*Cuscuta reflexa*	*Cre*	NC_009766
30	*Cucumis sativus*	*Csa*	NC_007144
31	*Citrus sinensis*	*Csi*	NC_008334
32	*Chloranthus spicatus*	*Csp*	NC_009598
33	*Cycas taitungensis*	*Cta*	NC_009618
34	*Chara vulgaris*	*Cvu*	NC_008097
35	*Crucihimalaya wallichii*	*Cwa*	NC_009271
36	*Daucus carota*	*Dca*	NC_008325
37	*Dioscorea elephantipes*	*Del*	NC_009601
38	*Drimys granadensis*	*Dgr*	NC_008456
39	*Draba nemorosa*	*Dne*	NC_009272
40	*Eucalyptus globulus subsp. globulus*	*Egl*	NC_008115
41	*Epifagus virginiana*	*Evi*	NC_001568
42	*Fagopyrum esculentum subsp. Ancestrale*	*Fes*	NC_010776
43	*Guizotia abyssinica*	*Gab*	NC_010601
44	*Gossypium barbadense*	*Gba*	NC_008641

45	*Gossypium hirsutum*	*Ghi*	NC_007944
46	*Glycine max*	*Gma*	NC_007942
47	*Helianthus annuus*	*Han*	NC_007977
48	*Huperzia lucidula*	*Hlu*	NC_006861
49	*Helicosporidium sp ex Simulium jonesii*	*Hsi*	NC_008100
50	*Hordeum vulgare subsp. vulgare*	*Hvu*	NC_008590
51	*Illicium oligandrum*	*Iol*	NC_009600
52	*Ipomoea purpurea*	*Ipu*	NC_009808
53	*Jasminum nudiflorum*	*Jnu*	NC_008407
54	*Lycopersicon esculentum cultivar LA3023*	*Les*	DQ347959
55	*Lotus japonicus*	*Lja*	NC_002694
56	*Lobularia maritima*	*Lma*	NC_009274
57	*Lemna minor*	*Lmi*	NC_010109
58	*Lolium perenne*	*Lpe*	NC_009950
59	*Lactuca sativa*	*Lsa*	NC_007578
60	*Leptosira terrestris*	*Lte*	NC_009681
61	*Liriodendron tulipifera*	*Ltu*	NC_008326
62	*Lepidium virginicum*	*Lvi*	NC_009273
63	*Manihot esculenta*	*Mes*	NC_010433
64	*Morus indica*	*Min*	NC_008359
65	*Marchantia polymorpha*	*Mpo*	NC_001319
66	*Medicago truncatula*	*Mtr*	NC_003119
67	*Mesostigma viride*	*Mvi*	NC_002186
68	*Nuphar advena*	*Nad*	NC_008788
69	*Nymphaea alba*	*Nal*	NC_006050
70	*Nandina domestica*	*Ndo*	NC_008336
71	*Nasturtium officinale*	*Nof*	NC_009275
72	*Nephroselmis olivacea*	*Nol*	NC_000927
73	*Nicotiana sylvestris*	*Nsy*	NC_007500
74	*Nicotiana tabacum*	*Nta*	NC_001879
75	*Nicotiana tomentosiformis*	*Nto*	NC_007602
76	*Oenothera argillicola*	*Oar*	NC_010358
77	*Oenothera biennis*	*Obi*	NC_010361
78	*Oedogonium cardiacum*	*Oca*	NC_011031
79	*Oenothera glazioviana*	*Ogl*	NC_010360
80	*Oryza nivara*	*Oni*	NC_005973
81	*Oenothera parviflora*	*Opa*	NC_010362
82	*Olimarabidopsis pumila*	*Opu*	NC_009267
83	*Oryza sativa (indica cultivar-group)*	*Osa*	AY522329
84	*Ostreococcus tauri*	*Ota*	NC_008289
85	*Oltmannsiellopsis viridis*	*Ovi*	NC_008099
86	*Pseudendoclonium akinetum*	*Pak*	NC_008114
87	*Populus alba*	*Pal*	NC_008235
88	*Phalaenopsis aphrodite subsp. formosana*	*Pap*	NC_007499
89	*Piper cenocladum*	*Pce*	NC_008457

90	*Panax ginseng*	*Pgi*	NC_006290
91	*Pelargonium x hortorum*	*Pho*	NC_008454
92	*Pinus koraiensis*	*Pko*	NC_004677
93	*Psilotum nudum*	*Pnu*	NC_003386
94	*Platanus occidentalis*	*Poc*	NC_008335
95	*Physcomitrella patens subsp. patens*	*Ppa*	NC_005087
96	*Pinus thunbergii*	*Pth*	NC_001631
97	*Populus trichocarpa*	*Ptr*	NC_009143
98	*Phaseolus vulgaris*	*Pvu*	NC_009259
99	*Ranunculus macranthus*	*Rma*	NC_008796
100	*Sorghum bicolor*	*Sbi*	NC_008602
101	*Solanum bulbocastanum*	*Sbu*	NC_007943
102	*Stigeoclonium helveticum*	*She*	NC_008372
103	*Saccharum hybrid cultivar SP-80-3280*	*Shy*	NC_005878
104	*Solanum lycopersicum*	*Sly*	NC_007898
105	*Scenedesmus obliquus*	*Sob*	NC_008101
106	*Saccharum officinarum*	*Sof*	NC_006084
107	*Spinacia oleracea*	*Sol*	NC_002202
108	*Staurastrum punctulatum*	*Spu*	NC_008116
109	*Solanum tuberosum*	*Stu*	NC_008096
110	*Selaginella uncinata*	*Sun*	AB197035
111	*Triticum aestivum*	*Tae*	NC_002762
112	*Trachelium caeruleum*	*Tca*	NC_010442
113	*Vitis vinifera*	*Vvi*	NC_007957
114	*Welwitschia mirabilis*	*Wmi*	NC_010654
115	*Zygnema circumcarinatum*	*Zci*	NC_008117
116	*Zea mays*	*Zma*	NC_001666
