# Supplementary material for: A comparative approach to elucidate chloroplast genome replication
Source: BMC Genomics. 2009 May 20;10:237. doi: 10.1186/1471-2164-10-237 (PMC2695485; doi:10.1186/1471-2164-10-237)
Supplement: Additional File 2 — Locations of complete and partial sequence homologues to the tobacco chloroplast replication origin sequences. Begin and end limits are shown in this table for replication origin homologues of each sub-category of genomes, for the replication origin as the query (Q) sequence and the complete chloroplast genome as the subject (S) sequence. Reverse complementary sequence homologues to any of the replication origins (A1, A2, B and R) are indicated as '-C'. Partial homologues to any replication origin sequence are represented as the abbreviation for that origin, superscripted with P. Blast analyses were performed using NCBI's pairwise blast tool . [file 1471-2164-10-237-S2.pdf]

Category I

A

Species	Region	B^1/B^1_C		$A2_C$		A1		B		B_C		$A1_C$		A2		B^1_C/B^1	
Aam	Q	187	218	82	1	1	35	23	242	242	23	35	1	1	82	218	187
	S	87660	87691	101536	101617	102297	102331	109079	109331	127985	128237	134985	135019	135699	135780	149625	149656
Aca	Q	57	25	82	1	1	35	221	2	2	221	35	1	1	82	26	57
	S	87985	88016	101861	101942	102622	102656	109404	109656	128315	128567	135315	135349	136029	136110	149955	149986

B

Species	Region	$A2_C$		A1		B^1		B		B_C		B^1_C		$A1_C$		A2	
Jnu	Q	82	1	1	35	15	55	162	196	196	162	55	15	35	1	1	82
	S	109761	109842	110523	110557	117243	117283	117315	117349	140650	140684	140716	140756	147442	147476	148157	148238
Lmi	Q	82	1	1	35	1	52	152	224	224	152	52	1	35	1	1	82
	S	108166	108247	108748	108782	115304	115355	115434	115506	140357	140429	140508	140559	147081	147115	147616	147697

Category II

C

Species	Region	$A2_C$		A1		B		B^1_C		B_C		$A1_C$		A2	
Pce	Q	82	1	1	35	1	201	201	131	201	1	35	1	1	82
	S	106633	106714	107397	107431	113996	114185	133502	133573	134108	134297	140862	140896	141579	141660

Category III

D

Species	Region	$A2_C$		A1		B		B_C		$A1_C$		A2	
Aco	Q	82	1	1	35	74	198	198	74	35	1	1	82
	S	102286	102367	103052	103086	109678	109796	127791	127909	134501	134535	135220	135301
Agr	Q	82	1	1	35	74	198	198	74	35	1	1	82
	S	102331	102412	103097	103131	109738	109856	127876	127994	134601	134635	135320	135401
Atr	Q	82	1	1	35	74	198	198	74	35	1	1	82
	S	102331	102412	103097	103131	109738	109856	127876	127994	134601	134635	135320	135401
Ath	Q	82	1	1	35	74	207	207	74	35	1	1	82
	S	103019	103100	103567	103601	110182	110309	128340	128467	135048	135082	135549	135630
Ahi	Q	82	1	1	35	74	207	207	74	35	1	1	82
	S	101603	101684	102368	102402	108990	109117	127392	127519	134107	134141	134825	134906
Abe	Q	82	1	1	35	1	243	243	1	35	1	1	82
	S	105079	105160	105614	105648	112083	112325	131232	131474	137909	137943	138397	138478
Bve	Q	82	1	1	35	74	207	207	74	35	1	1	82
	S	102280	102361	103046	103080	109685	109812	128156	128283	134888	134922	135607	135688
Bmi	Q	82	1	1	35	1	219	219	1	35	1	1	82
	S	106885	106966	107651	107685	114251	114463	132692	132904	139470	139504	140189	140270
Cbu	Q	82	1	1	35	74	207	207	74	35	1	1	82
	S	102568	102649	103335	103369	109948	110075	128157	128284	134863	134897	135583	135664
Cpa	Q	82	1	1	35	73	201	201	73	35	1	1	82

	S	107542	107624	108311	108345	114918	115049	133801	133932	140505	140539	141226	141308
Csi	Q	82	1	1	35	31	210	210	31	35	1	1	82
	S	107064	107145	107846	107880	114421	114594	133280	133453	139994	140028	140729	140810
Car	Q	82	1	1	35	1	243	243	1	35	1	1	82
	S	103339	102412	104099	104133	110708	110944	129412	129648	136223	136257	136935	137017
Cwa	Q	82	1	1	35	74	207	207	74	35	1	1	82
	S	102978	103059	103744	103778	110364	110491	128806	128933	135553	135519	136238	136319
Csa	Q	82	1	1	35	1	187	187	1	35	1	1	82
	S	104236	104317	104871	104905	111537	111718	130192	130372	137004	137038	137592	137673
Cre	Q	82	1	1	35	146	124	50	14	35	1	1	82
	S	90106	90186	90869	90903	103961	103983	104009	104045	110087	110121	110804	110884
Dca	Q	82	1	1	35	6	224	224	6	35	1	1	82
	S	103051	103132	103822	103856	110361	110573	129581	129793	136298	136332	137022	137103
Dne	Q	82	1	1	35	74	207	207	74	35	1	1	82
	S	101179	101260	101946	101980	108572	108699	127064	127191	133783	133817	134503	134584
Fes	Q	82	1	1	35	1	229	229	1	35	1	1	82
	S	102990	103071	103755	103789	110542	110764	133719	133941	140694	140728	141412	141493
Gba	Q	82	1	1	35	145	120	57	11	35	1	1	82
	S	107681	107772	108469	108503	133918	133943	133994	134040	140656	140690	141387	141468
Ghi	Q	82	1	1	35	11	57	120	145	35	1	1	82
	S	107661	107742	108439	108473	115095	115141	115192	115217	140624	140658	141355	141436
Lvi	Q	82	1	1	35	74	125	125	74	35	1	1	82
	S	102846	102927	103612	103646	110275	110326	128453	128504	135133	135167	135852	135933
Les	Q	82	1	1	35	1	243	243	1	35	1	1	82
	S	103983	104064	104524	104558	111115	111357	129981	130223	136780	136814	137274	137355
Mes	Q	82	1	1	35	61	241	241	61	35	1	1	82
	S	108248	108329	108978	109012	115633	115807	134902	135076	141697	141731	142380	142461
Min	Q	82	1	1	35	6	169	169	6	35	1	1	82
	S	105520	105601	106283	106317	112796	112947	132924	133075	139554	139588	140270	140351
Nof	Q	82	1	1	35	74	207	207	74	35	1	1	82
	S	103129	103210	103896	103930	110275	110649	128732	128859	135451	135485	135852	135933
Nsy	Q	82	1	1	35	1	243	243	1	35	1	1	82
	S	104769	104850	105295	105329	111778	112020	130606	130850	137297	137331	137776	137857
Nta	Q	82	1	1	35	1	243	243	1	35	1	1	82
	S	104771	104852	105297	105331	111780	112022	130608	130850	137299	137333	137778	137859
Nto	Q	82	1	1	35	1	243	243	1	35	1	1	82
	S	104541	104622	105076	105110	111560	111802	130336	130578	137028	137062	137516	137597
Nad	Q	82	1	1	35	205	127	111	1	35	1	1	82
	S	109210	109292	109983	110017	134535	134613	134617	134717	141229	141263	141954	142036
Opu	Q	82	1	1	35	74	207	207	74	35	1	1	82
	S	102730	102811	103495	103529	110119	110246	128375	128502	135092	135126	135810	135891
Ptr	Q	82	1	1	35	13	241	241	13	35	1	1	82
	S	104401	104482	105168	105202	111833	112055	130108	130330	136961	136995	137681	137762
Rma	Q	82	1	1	35	2	111	131	220	35	1	1	82
	S	102998	103080	103766	103800	110987	111096	111104	111199	135968	136002	136688	136770
Sbu	Q	82	1	1	35	1	243	243	1	35	1	1	82
	S	103861	103942	104402	104436	111002	111244	129884	130126	136692	136726	137186	137267

Sly	Q	82	1	1	35	1	243	243	1	35	1	1	82
	S	103986	104067	104527	104561	111118	111360	129984	130226	136783	136817	137277	137358
Stu	Q	82	1	1	35	1	243	243	1	35	1	1	82
	S	103814	103895	104355	104389	110956	111198	129838	130080	136647	136681	137141	137222
Sol	Q	82	1	1	35	23	224	224	23	35	1	1	82
	S	99955	100036	100507	100541	107098	107293	126152	126347	132924	132938	133409	133490
Vvi	Q	82	1	1	35	1	224	224	1	35	1	1	82
	S	107802	107883	108570	108604	115133	115350	134710	134927	141456	141490	142177	142258
Pal	Q	82	1	1	35	13	241	241	13	35	1	1	82
	S	103900	103981	104667	104701	111332	111554	129570	129792	136423	136457	137143	137224
Dgr	Q	82	1	1	35	2	217	217	2	35	1	1	82
	S	107638	107720	108409	108443	115069	115272	134018	134221	140847	140881	141570	141652
Egl	Q	82	1	1	35	1	228	228	1	35	1	1	82
	S	107683	107764	108455	108489	115063	115278	134021	134236	140810	140844	141535	141616
Csp	Q	82	1	1	35	1	223	223	1	35	1	1	82
	S	105300	105381	106074	106108	112587	112797	131964	132174	138653	138687	139380	139461
Pgi	Q	82	1	1	35	3	243	243	3	35	1	1	82
	S	103939	104020	104703	104737	111286	111672	130753	131139	137688	137722	138405	138486

E

**Species**	**Region**	**$A2_C$**		**A1**		**B**		**B_C**		**A2**		**$A1_C$**	
Lma	Q	82	1	1	35	74	207	207	74	1	82	35	1
	S	101054	1011[illegible]	107596	10763[illegible]	108443	108570	126489	126616	133924	134005	140407	140441

F

**Species**	**Region**	**$A2_C$**		**A1**		**B**		**B^1**		**B_C**		**$A1_C$**	
Ndo	Q	82	1	1	35	1	111	127	241	111	1	35	1
	S	104081	104163	104847	104881	111402	111512	111534	111649	130561	130671	137192	137226

Category IV

G

**Species**	**Region**	**$A2_C$**		**A1**		**B_C/B**		**$A1_C$**		**A2**	
Iol	Q	82	1	1	35	241	1	35	1	1	82
	S	106116	106197	106875	106909	132878	133106	139702	139736	140414	140495
Cfe	Q	82	1	1	35	99	2	35	1	1	82
	S	103403	103485	104180	104214	129461	129558	136068	136102	136797	136879
Cfl	Q	82	1	1	35	99	2	35	1	1	82
	S	103403	103485	104180	104214	129461	129558	136068	136102	136797	136879
Cex	Q	82	1	1	35	50	14	35	1	1	82
	S	93303	93383	94066	94100	107839	107875	113995	114029	114172	114792
Nal	Q	82	1	1	35	111	2	35	1	1	82
	S	108578	108660	109351	109385	134503	134162	140560	140594	141285	141367
Pap	Q	82	1	1	35	219	125	35	1	1	82
	S	105178	105258	105941	105975	122277	122371	128947	128981	129664	129744
Poc	Q	82	1	1	35	241	2	35	1	1	82

	S	110301	110383	111062	111096	136063	136296	142846	142880	143559	143641
Del	Q	82	1	1	35	1	117	35	1	1	82
	S	101370	101452	102137	102171	108676	108786	133216	133250	133935	134017

Category V

H

Species	Region	A1$_C$		A2		A2$_C$		A1	
Acav	Q	35	1	32	82	82	32	1	35
	S	90056	90090	90844	90894	141957	142007	142761	142795
Pho	Q	35	1	32	82	82	32	1	35
	S	84294	84238	85022	85103	192550	192631	193325	193359

I

Species	Region	A2$_C$		A1		A1$_C$		A2	
Ast	Q	82	1	1	35	35	1	1	82
	S	94785	94867	95406	95440	122757	122791	123330	123412
Aev	Q	70	33	1	35	35	1	33	70
	S	104351	104388	105092	105126	138485	138519	139223	139260
Afo	Q	82	32	1	35	35	1	32	82
	S	115514	115564	116281	116315	152351	152385	153102	153152
Ami	Q	82	32	1	35	35	1	32	82
	S	80087	80135	80782	80816	104745	104779	105426	105474
Bdi	Q	82	1	1	35	35	1	1	82
	S	93045	93126	93665	93699	120948	120982	121521	121602
Cde	Q	82	1	1	35	35	1	1	82
	S	104809	104890	105577	105611	137249	137283	137970	138051
Cta	Q	82	27	1	35	35	1	27	82
	S	107761	107816	108513	108547	144715	144749	145446	145501
Gab	Q	82	1	1	35	35	1	1	82
	S	101652	101733	102247	102281	132999	133033	133549	133630
Gma	Q	82	1	1	35	35	1	1	82
	S	101612	101693	102384	102418	132976	133010	133701	133782
Han	Q	82	1	1	35	35	1	1	82
	S	101226	101307	101824	101858	132777	132811	133328	133409
Hvu	Q	82	1	1	35	35	1	1	82
	S	94706	94788	95327	95361	122773	122807	123346	123428
Hlu	Q	65	25	1	35	35	1	25	65
	S	109059	109099	109811	109845	148617	148651	149363	149403
Ipu	Q	82	1	1	35	35	1	1	82
	S	103842	103922	104591	104625	145594	145628	146297	146377
Lsa	Q	82	1	1	35	35	1	1	82
	S	102198	102279	102964	102998	133871	133905	134590	134761
Lpe	Q	82	1	1	35	35	1	1	82
	S	93491	93573	94113	94147	121100	121134	121674	121756

Lja	Q	82	1	1	35	35	1	1	82
	S	100242	100323	100782	100816	131640	131674	132133	132214
Oar	Q	82	1	1	35	35	1	1	82
	S	108587	108667	109353	109387	144180	144214	144900	144980
Obi	Q	82	1	1	35	35	1	1	82
	S	108740	108820	109506	109540	144232	144266	144952	145032
Ogl	Q	82	1	1	35	35	1	1	82
	S	109272	109352	110038	110072	144745	144779	145465	145545
Oni	Q	82	1	1	35	35	1	1	82
	S	93270	93350	94032	94066	120973	121007	121689	121769
Opa	Q	82	1	1	35	35	1	1	82
	S	107406	107486	108172	108206	142892	142926	143612	143692
Osa	Q	82	1	1	35	35	1	1	82
	S	93275	93355	94037	94071	120979	121013	121695	121775
Pvu	Q	82	1	1	35	35	1	1	82
	S	99170	99250	99983	99967	130142	130176	130859	130939
Shy	Q	82	1	1	35	35	1	1	82
	S	18328	18410	19094	19128	45590	46024	46708	46790
Sof	Q	82	1	1	35	35	1	1	82
	S	97885	97967	98651	98685	125546	125580	126264	126346
Sbi	Q	82	1	1	35	35	1	1	82
	S	98051	98134	98818	98852	125636	125670	126354	126437
Tae	Q	82	1	1	35	35	1	1	82
	S	93072	93154	93693	93727	121167	121201	121740	121822
Tca	Q	82	1	1	35	35	1	1	82
	S	102983	103064	103730	103764	158668	158702	159368	159449
Wmi	Q	82	27	1	35	35	1	27	82
	S	81848	81903	82579	82613	105670	105704	106380	106435
Zma	Q	82	1	1	35	35	1	1	82
	S	97174	97256	97939	97973	124764	124798	125481	125563
Evi	Q	82	1	1	30	30	1	1	82
	S	35600	35672	36331	36360	53468	53497	54156	54228

J

**Species**	**Region**	**A2$_{C}$**		**B**		**B$_{C}$**		**A2**	
Ltu	Q	82	1	1	170	170	1	1	82
	S	106831	106912	114164	114321	133716	133873	141125	141206

Category VI

K

**Species**	**Region**	**A1**		**A1$_{C}$**	
Chgl	Q	1	35	35	1
	S	93689	93723	126143	126177
Cvu	Q	1	35	35	1
	S	140177	140211	180538	180572

Cat	Q	1	35	35	1
	S	111413	111447	149906	149940
Mvi	Q	1	35	35	1
	S	86133	86167	115821	115855
Pnu	Q	1	35	35	1
	S	93912	93946	129501	129535
Sob	Q	12	35	35	12
	S	77069	77092	156800	156823
Ota	Q	1	35	35	1
	S	37271	37305	69632	69666
Ppa	Q	1	35	35	1
	S	88887	88921	119181	119215

L

Species	Region	$A2_C$		A2	
Mpo	Q	55	1	1	55
	S	84097	84151	117969	118023

M

Species	Region	$A1_C$		A2	
Mtr	Q	35	1	1	82
	S	24818	24852	25279	25360

N

Species	Region	$A2_C$		A1	
Pth	Q	82	29	1	35
	S	86427	86479	87228	87262
Pko	Q	82	1	1	35
	S	84841	84931	85646	85680

Category VII

O

Species	Region	$A1_C$/A1	
Cja	Q	35	1
	S	104617	104651
Lte	Q	5	35
	S	194733	194763
Spu	Q	1	35
	S	26788	26822
She	Q	1	35
	S	223510	223544
Zci	Q	1	35
	S	132343	132377
