# Supplementary material for: A comparative approach to elucidate chloroplast genome replication
Source: BMC Genomics. 2009 May 20;10:237. doi: 10.1186/1471-2164-10-237 (PMC2695485; doi:10.1186/1471-2164-10-237)
Supplement: Additional File 3 — t-statistics of the regression slopes between (A/A+G) ratios at each site and their relative positions on the genome, in regions between homologues to tobacco chloroplast replication origin sequences. The t-statistics of the A → G (i.e. A/(A+G)) deamination trends (i.e. of regression slopes between A/A+G ratios at each site and the relative position of this site on the genome) are depicted for all sub-categories of genomes in this table, for the complete regions between homologues to tobacco chloroplast replication origins (first row for each species appended with '-C'), and after dividing these regions mid-way, for the first half (second row for each species appended with '-I') and second half (third row for each species appended with '-II') respectively for these regions. Significant values (p < 0.05, 2-tailed t-test) are emboldened. [file 1471-2164-10-237-S3.pdf]

Category I

A

**Species**	**Len**	**$A2_C$->A1**	**A1->B**	**B->B_C**	**B_C->$A1_C$**	**A1->$A1_C$**	**$A1_C$->A2**	**A2->$A2_C$**
Aam-C	153819	-1.52	**8.21**	-0.66	**-5.37**	0.34	-1.51	**-4.95**
Aam-I		-0.04	-0.78	**-3.18**	**-6.87**	**16.19**	-0.80	**9.65**
Aam-II		0.03	**7.6**	1.91	**2.9**	**-14.96**	-1.18	**-11.49**
Aca-C	153821	-1.52	**8.21**	-0.68	**-5.34**	0.54	-1.51	**-3.54**
Aca-I		-0.04	-0.70	**-3.21**	**-6.78**	**16.34**	-0.80	**9.26**
Aca-II		0.03	**7.65**	1.9	**2.94**	**-15.03**	-1.18	**-10.96**
B								
Jnu-C	165121	-1.20	**5.54**	**-3.34**	**-4.73**	-0.76	-1.36	**-7.13**
Jnu-I		-0.35	-0.19	**3.32**	**-5.39**	**19.34**	-0.85	**6.62**
Jnu-II		0.03	**6.37**	**-4.42**	**2.55**	**-15.79**	-0.77	**-11.6**
Lmi-C	165955	-0.97	**7.19**	**-2.81**	**-6.44**	-0.29	-0.95	**-5.57**
Lmi-I		-0.58	-0.36	**3.33**	**-6.58**	**10.82**	0.84	**9.79**
Lmi-II		1.00	**7.65**	**-7.77**	**2.61**	**-18.69**	-1.61	**-14.7**

Category II

C

**Species**	**Len**	**$A2_C$->A1**	**A1->B**	**B->B_C**	**B_C->$A1_C$**	**A1->$A1_C$**	**$A1_C$->A2**	**A2->$A2_C$**
Pce-C	160624	-0.98	**5.81**	1.14	**-3.56**	0.34	-1.35	**-6.11**
Pce-I		-0.39	-0.77	-1.34	**-3.96**	**16.23**	-1.08	**6.96**
Pce-II		-0.08	**6.64**	-0.59	**2.35**	**-17.08**	-1.29	**-12.34**

Category III

D

**Species**	**Len**	**$A2_C$->A1**	**A1->B**	**B->B_C**	**B_C->$A1_C$**	**A1->$A1_C$**	**$A1_C$->A2**	**A2->$A2_C$**
Aco-C	154168	-1.12	**5.57**	**-4.17**	**-3.38**	-0.42	-0.98	**-6.79**
Aco-I		-0.32	-0.23	**-3.12**	**-4.62**	**21.11**	-0.63	**11.48**
Aco-II		-0.3	**6.54**	-0.25	**3.58**	**-18.19**	-0.71	**-14.32**
Agr-C	154243	-0.92	**5.46**	**-3.76**	**-3.35**	-0.38	-1.16	**-6.62**
Agr-I		-0.19	-0.24	**-3.53**	**-4.64**	**20.45**	-0.66	**10.99**
Agr-II		-0.42	**6.52**	-0.84	**3.61**	**-18.33**	-0.67	**-14.08**
Atr-C	162686	-0.74	**5.32**	-0.75	**-2.11**	-0.24	-1.23	**-8.20**
Atr-I		0.98	-0.5	-0.79	**-3.78**	**15.7**	-1.25	**6.07**
Atr-II		-0.17	**6.55**	0.32	**2.95**	**-16.49**	-1.31	**-10.96**
Ath-C	154478	-0.94	**4.92**	-1.72	**-3.43**	0.48	-0.76	**-7.52**
Ath-I		-0.87	-0.04	**-2.24**	**-5.05**	**20.53**	0.55	**11.83**
Ath-II		0.52	**6.54**	0.03	**3.19**	**-18.58**	-1.14	**-15.24**
Ahi-C	153689	-0.95	**5.39**	**-2.43**	**-3.52**	0.1	-1.09	**-6.91**
Ahi-I		-0.41	-0.25	**-3.13**	**-4.7**	**21.01**	-0.66	**12**
Ahi-II		-0.13	**6.62**	0.57	**3.35**	**-18.96**	-0.73	**-15.07**
Abe-C	156687	-1.4	**4.78**	-0.34	**-3.25**	0.34	-0.86	**-6.52**

Abe-I		-1.38	-0.47	-1.82	**-4.37**	**17.09**	0.28	**8.36**
Abe-II		0.88	**6.05**	0.83	**2.4**	**-17.75**	-1.15	**-13.99**
Bve-C	154532	-0.84	**5.61**	**-2.00**	**-3.38**	-0.04	-1.05	**-7.25**
Bve-I		-0.38	-0.27	**-3.52**	**-4.67**	**20.85**	-0.66	**11.12**
Bve-II		-0.13	**6.87**	-0.32	**3.43**	**-19.81**	-0.49	**-14.8**
Bmi-C	159010	-1.43	0.46	-0.80	-0.78	0.42	-1.22	**-4.83**
Bmi-I		-0.14	-0.18	-0.94	-0.63	**16.17**	-0.65	1.32
Bmi-II		-0.11	-0.77	1.16	-0.77	**-17.42**	-1.26	**-3.03**
Cbu-C	154490	-0.85	**5.43**	**-2.26**	**-3.18**	-0.08	-0.36	**-7.21**
Cbu-I		-0.26	-0.22	**-2.27**	**-4.72**	**20.52**	0.56	**12.23**
Cbu-II		0.22	**6.79**	0.14	**3.22**	**-18.45**	-0.85	**-13.97**
Cpa-C	160100	-1.59	**6**	-1.45	**-3.58**	0.02	-0.86	**-7.85**
Cpa-I		-0.59	-0.49	**-2.43**	**-4.95**	**18.88**	-0.61	**9.03**
Cpa-II		-0.32	**6.17**	1.82	**3.33**	**-18**	-0.66	**-15.7**
Csp-C	157772	-0.96	**3.74**	0.68	**-2.69**	1.66	-1.38	**-6.27**
Csp-I		-0.16	0.24	**-2.15**	**-4.13**	**12.24**	-0.93	**6.97**
Csp-II		-0.27	**5.7**	1.19	**2.91**	**-14**	-1.18	**-10.67**
Csi-C	160129	-1.83	**4.42**	-1.29	**-3.45**	0.22	-0.38	**-6.53**
Csi-I		-0.8	-0.2	**-3.58**	**-5.04**	**15.74**	-0.44	**8.1**
Csi-II		0.07	**6.31**	1.17	**3.13**	**-15.95**	-0.71	**-11.68**
Car-C	155189	-1.15	**5.96**	-0.14	**-3.52**	-0.57	-1.21	**-6.96**
Car-I		-0.32	-0.69	**-2.58**	**-4.18**	**17.12**	-0.52	**8.15**
Car-II		-0.26	**6.18**	0.2	**2.48**	**-19.75**	-0.91	**-15.68**
Cwa-C	155199	-0.81	**5.55**	**-2.71**	**-3.49**	-0.32	0.10	**-6.46**
Cwa-I		-0.07	-0.21	**-3.01**	**-4.86**	**21.23**	1.17	**11.66**
Cwa-II		0.11	**6.76**	0.04	**3.1**	**-19.15**	-0.79	**-14.48**
Csa-C	155293	-1.53	**5.29**	-1.27	**-3.47**	0.03	-1.14	**-8.01**
Csa-I		-0.39	-0.5	**-2.57**	**-4.56**	**18.18**	0.67	**9.89**
Csa-II		0.24	**6.65**	1.74	**2.79**	**-17.49**	-0.59	**-14.77**
Cre-C	121521	-1.74	**12.98**	-1.14	**-5.24**	0.68	-1.13	**-5.88**
Cre-I		-0.64	**6.76**	-0.71	**-6.23**	**13.86**	1.02	**7.9**
Cre-II		-0.28	**-3.88**	-0.89	**2.68**	**-10.88**	-1.44	**-14.98**
Dca-C	155911	-1.46	**5.73**	-0.70	**-3.42**	-0.18	-0.93	**-6.56**
Dca-I		-0.49	-0.46	**-2.95**	**-4.86**	**17.28**	-0.65	**8.97**
Dca-II		0.42	**6.81**	-1.37	**2.93**	**-19.64**	-1.09	**-13.91**
Dne-C	153289	-0.84	**5.10**	**-2.25**	**-3.31**	-0.1	-1.14	**-7.57**
Dne-I		-0.4	-0.54	**-3.21**	**-4.63**	20.91	-0.65	**8.69**
Dne-II		-0.4	**6.66**	0.68	**3.44**	-19.26	-0.61	**-14.07**
Dgr-C	160604	-1.1	**6.90**	1.11	**-3.60**	0.3	-1.38	**-6.09**
Dgr-I		-0.12	-1.04	**-4.43**	**-4.79**	**13.95**	-0.92	**4.66**
Dgr-II		-0.13	**5.94**	0.9	**2.85**	**-16.92**	-0.7	**-10.35**
Egl-C	160286	-1.22	**6.06**	-1.30	**-3.69**	-0.89	-1.2	**-7.68**
Egl-I		0	-0.76	**-6.15**	**-4.42**	**17.72**	-0.52	**7.72**
Egl-II		0.15	**6.86**	0.35	**2.94**	**-19.2**	-0.58	**-15.91**
Gba-C	160317	-1.32	**12.88**	1.19	**-5.58**	-0.73	-1.1	**-7.63**
Gba-I		-0.61	**16.73**	-0.21	**-6.22**	**18.8**	-0.49	**9.41**
Gba-II		-0.32	**-4.99**	0.52	**2.93**	**-14.07**	-0.7	**-15.67**

Ghi-C	160301	-1.32	**3.83**	**2.2**	**-16.00**	1.02	-1.1	**-7.71**
Ghi-I		-0.61	-0.26	0.4	-1.62	**10.48**	-0.49	**8.74**
Ghi-II		-0.32	**6.06**	0.7	**-15.54**	**-15.71**	-0.7	**-15.96**
Lvi-C	154743	-0.77	**5.45**	**-2.34**	**-3.48**	-0.02	-0.36	**-7.01**
Lvi-I		-0.07	-0.31	**-3.2**	**-4.66**	**20.71**	0.55	**11.25**
Lvi-II		0.08	**6.44**	-0.18	**3.32**	**-18.98**	-0.85	**-14.75**
Les-C	155461	-1.42	**5.91**	0.26	**-3.14**	0.12	-0.87	**-6.85**
Les-I		-0.99	-0.56	**-2.27**	**-4.02**	**16.58**	0.28	**7.42**
Les-II		0.66	**6.48**	1.56	**2.72**	**-18.34**	-1.14	**-13.59**
Mes-C	161453	-0.99	**6.24**	-1.12	**-3.86**	-0.05	-1.02	**-4.21**
Mes-I		-0.91	-0.91	**-2.66**	**-5.08**	**20.1**	-0.49	**14.65**
Mes-II		0.61	**6.73**	-0.34	**2.94**	**-20.9**	-0.64	**-16.79**
Min-C	158484	-1.82	**5.7**	**-2.35**	**-2.89**	-0.47	-1.27	**-7.26**
Min-I		-0.64	-0.7	**-4.35**	**-4.43**	**20.6**	-0.62	**10.42**
Min-II		-0.1	**6.62**	1.43	**3.43**	**-19.65**	-0.59	**-15.77**
Ndo-C	156599	-1.47	**6.01**	0.24	0.41	0.28	**-3.82**	**-4.83**
Ndo-I		-0.64	-0.75	0.22	**-3.13**	**16.35**	**-4.85**	**9.14**
Ndo-II		0.21	**6.4**	0.43	1.26	**-18.75**	**2.94**	**-12.21**
Nof-C	155105	-1.01	**5.47**	**-2.39**	**-3.40**	-0.01	-1.04	**-7.39**
Nof-I		-0.52	-0.19	**-2.92**	**-4.75**	**20.67**	-0.76	**11.5**
Nof-II		-0.08	**6.83**	-0.36	**3.46**	**-19.22**	-0.61	**-14.99**
Nsy-C	155941	-1.60	**5.83**	-0.33	**-3.06**	0.16	-1.35	**-7.34**
Nsy-I		-1.66	-0.52	**-2.04**	**-4.33**	**16.66**	0.76	**7.78**
Nsy-II		-0.7	**6.47**	1.15	**2.61**	**-17.4**	-0.51	**-13.52**
Nta-C	155943	-1.55	**5.83**	**-5.82**	**-5.06**	0.62	-0.83	**-7.02**
Nta-I		-1.84	-0.55	-1.53	**-5.26**	**18.19**	0.06	**7.96**
Nta-II		0.41	-0.55	**-3.56**	**2.93**	**-14.94**	-1.44	**-13.52**
Nto-C	155745	-1.38	**5.87**	-0.99	**-3.01**	-0.03	-0.86	**-7.17**
Nto-I		-1.18	-0.47	**-2.2**	**-4.2**	**16.86**	0.28	**7.83**
Nto-II		0.71	**6.4**	0.57	**2.62**	**-17.42**	-1.15	**-13.73**
Nad-C	160866	-1.23	**12.72**	**-2.83**	**-5.12**	0.76	-1.27	**-5.99**
Nad-I		0.08	**13.2**	-	**-6.16**	**15.59**	-1.28	**6.95**
Nad-II		0.38	**-3.12**	**-2.83**	**2.82**	**-12.74**	-1.23	**-9.69**
Opu-C	154737	-0.81	**5.39**	**-2.19**	**-3.28**	0.2	-1.04	**-6.93**
Opu-I		-0.5	-0.21	**-2.52**	**-4.75**	**20.83**	-0.73	**10.98**
Opu-II		-0.19	**6.63**	-0.62	**3.31**	**-19.31**	-0.5	**-14.42**
Pgi-C	156318	-1.57	**6.02**	-1.21	**-2.65**	-0.54	-1.09	**-6.99**
Pgi-I		-0.7	-0.31	**-3.17**	**-4.57**	**16.98**	-0.52	**7.8**
Pgi-II		-0.25	**6.57**	-0.78	**3.03**	**-19.91**	-0.73	**-12.96**
Pal-C	156505	-1.44	**6.43**	0.60	**-4.85**	0.77	-1.06	**-6.06**
Pal-I		-0.38	-0.7	-0.71	**-5.25**	**18.34**	-0.45	**8.52**
Pal-II		-0.2	**7.02**	0.95	**2.61**	**-19.31**	-0.6	**-15.11**
Ptr-C	157033	-1.42	**6.4**	1.02	**-4.90**	-0.42	-1.06	**-6.55**
Ptr-I		-0.23	-0.95	-1	**-5.23**	**14.84**	-0.45	**8.52**
Ptr-II		-0.33	**7.04**	-0.17	**2.66**	**-14.82**	-0.6	**-15.21**
Rma-C	155129	-1.61	**8.16**	-	**-15.70**	0.76	-1.19	**-7.22**
Rma-I		-0.35	-1.14	-	-0.53	**17.01**	-0.75	**8.13**

Rma-II		-0.05	**6.5**	-	**-16.33**	**-15.37**	-0.72	**-13.84**
Sbu-C	155371	-1.42	**6.02**	0.62	**-3.24**	-0.12	-0.87	**-7.14**
Sbu-I		-0.99	-0.6	**-3.85**	**-4.11**	**15.85**	0.28	**5.87**
Sbu-II		0.66	**6.51**	0.92	**2.8**	**-17.89**	-1.14	**-12.97**
Sly-C	155461	-1.44	**4.69**	1.04	**-3.14**	0.19	-0.87	**-7.33**
Sly-I		-1.18	0.14	**-3.48**	**-4.02**	**16.12**	0.28	**5.98**
Sly-II		0.83	**6.53**	1.38	**2.72**	**-17.42**	-1.14	**-13.14**
Stu-C	155298	-1.44	**6.14**	0.31	**-3.24**	-0.09	-0.87	**-7.30**
Stu-I		-1.18	-0.4	**-3.78**	**-4.16**	**16.04**	0.28	**5.92**
Stu-II		0.83	**6.48**	1.03	**2.76**	**-17.4**	-1.14	**-13.15**
Sol-C	150725	-0.91	**5.7**	-1.23	**-2.22**	-0.72	-1.08	**-7.83**
Sol-I		-0.86	-1	**-3.07**	**-3.84**	**20.14**	0.65	**9.98**
Sol-II		1.17	**6.62**	1.63	**3.11**	**-19.6**	-0.96	**-15.26**
Vvi-C	160928	-1.56	**6.05**	0.03	**-3.27**	-0.08	-0.97	**-7.71**
Vvi-I		-0.38	-0.6	**-5.57**	**-4.7**	**16.29**	-0.73	**7.81**
Vvi-II		-0.14	**6.75**	0.18	**2.99**	**-18.95**	-1.11	**-12.7**

E

Lma-C	152659	**3.05**	0.39	-1.58	**-1.96**	-0.87	**7.48**	**-12.48**
Lma-I		-1.52	-0.73	**-2.6**	**-5.51**	**21.22**	**6.31**	**3.28**
Lma-II		**4.8**	0.58	0.42	**4.06**	**-5.3**	-0.12	**-13.44**

Category IV

**Species**	**Len**	**$A2_C$->A1**	**A1->B**	**B->B_C**	**B_C->$A1_C$**	**A1->$A1_C$**	**$A1_C$->A2**	**A2->$A2_C$**
Cex-C	125373	-1.74	-	-	**-4.16**	-0.76	-1.15	**-8.58**
Cex-I		-0.83	-	-	**-5.62**	**15.36**	0.49	**6.38**
Cex-II		0.2	-	-	**3.04**	**-13.08**	-1.49	**-15.37**
Cfe-C	153337	-0.98	-	-	**-3.19**	-0.68	-1.55	**-5.19**
Cfe-I		-0.11	-	-	**-4.66**	**15.27**	-1.05	**5.35**
Cfe-II		-0.62	-	-	**2.93**	**-16.39**	-1.04	**-8.74**
Cfl-C	153337	-0.98	-	-	**-3.19**	-0.68	-1.55	**-5.19**
Cfl-I		-0.11	-	-	**-4.66**	**15.27**	-1.05	**5.35**
Cfl-II		-0.62	-	-	**2.93**	**-16.39**	-1.04	**-8.74**
Del-C	152609	-1.15	-	-	**-16.79**	**1.99**	-1.26	**-6.03**
Del-I		0.17	-	-	**-2.26**	**14.25**	-0.7	**6.94**
Del-II		0.16	-	-	**-14.3**	**-16.69**	-0.89	**-14.02**
Iol-C	148553	-1.11	-	-	**-3.54**	-0.14	-1.4	**-6.31**
Iol-I		-0.1	-	-	**-4.68**	**14.8**	-0.56	**4.8**
Iol-II		0.06	-	-	**3.1**	**-14.96**	-1.04	**-9.48**
Nal-C	159930	-1.2	-	-	**-3.08**	0.08	-0.71	**-5.87**
Nal-I		-0.07	-	-	**-5.11**	**14.62**	0.67	**6.89**
Nal-II		0.37	-	-	**3.05**	**-14.35**	-0.08	**-9.78**
Pap-C	148964	-0.4	-	-	**-5.84**	1.27	**-2.02**	**-5.29**
Pap-I		-0.54	-	-	**-6.42**	**18.79**	-0.77	**11.52**
Pap-II		0.24	-	-	**2.51**	**-15.42**	-1.43	**-14.95**

Poc-C	161791	-0.96	-	-	**-3.73**	-0.12	-1.4	**-7.26**
Poc-I		-0.12	-	-	**-4.99**	**15.1**	-0.7	**4.99**
Poc-II		0.28	-	-	**2.82**	**-17.13**	-0.75	**-13.15**

Category V

Species	Len	$A2_C$->A1	A1->B	B->B_C	B_C->$A1_C$	A1->$A1_C$	$A1_C$->A2	A2->$A2_C$
Acav-C	*150568*	-1.36	-	-	-	-1.1	0.88	-1.48
Acav-I		-1.77	-	-	-	**11.58**	0.8	**9.94**
Acav-II		0.87	-	-	-	**-11.6**	1.1	**-7.41**
Aev-C	*153901*	-0.27	-	-	-	0.7	-0.56	**-7.45**
Aev-I		0.86	-	-	-	**17.52**	-0.54	**7.43**
Aev-II		-0.35	-	-	-	**-14.48**	0.32	**-10.07**
Afo-C	*161162*	-0.62	-	-	-	**2.1**	-1.5	-1.84
Afo-I		1.72	-	-	-	**19.16**	-0.12	**5.77**
Afo-II		0.78	-	-	-	**-17.95**	0	**-10.51**
Ast-C	*136584*	-1.02	-	-	-	1.11	-1.39	**-3.17**
Ast-I		0.38	-	-	-	**16.26**	0.13	**9.75**
Ast-II		-0.67	-	-	-	**-15.21**	0.23	**-12.16**
Cde-C	*156252*	-1.26	-	-	-	-0.78	-1.49	**-6.84**
Cde-I		0.04	-	-	-	**16.41**	-1.46	**6.62**
Cde-II		0.32	-	-	-	**-14.95**	-0.96	**-13**
Cta-C	*163403*	-0.62	-	-	-	0.82	-1.55	**-6.57**
Cta-I		0.43	-	-	-	**16.4**	-1.42	**3.41**
Cta-II		1.27	-	-	-	**-14.56**	-0.57	**-6.32**
Evi-C	*70028*	**-2.22**	-	-	-	-0.44	-0.93	**-5.39**
Evi-I		-0.66	-	-	-	**14.68**	0.11	**9.93**
Evi-II		-0.11	-	-	-	**-6.92**	-0.18	**-14.1**
Gma-C	*152218*	-1.46	-	-	-	0.44	-0.79	**-5.19**
Gma-I		-0.15	-	-	-	**16.7**	-0.59	**8.19**
Gma-II		-0.14	-	-	-	**-16.49**	-0.57	**-15.81**
Han-C	*151104*	-1.58	-	-	-	1.34	-0.85	**-6.22**
Han-I		-0.74	-	-	-	**12.32**	0.74	**5.24**
Han-II		0.06	-	-	-	**-15.54**	-1.43	**-14.1**
Hlu-C	*154373*	-0.34	-	-	-	**18.81**	-1.91	**-2.78**
Hlu-I		-0.64	-	-	-	**14.14**	-1.61	**-5.35**
Hlu-II		0.65	-	-	-	**2.09**	0.32	**-6.79**
Hvu-C	*136462*	-1.47	-	-	-	0.81	-1.03	**-4.95**
Hvu-I		-0.12	-	-	-	**17.29**	0.38	**9.08**
Hvu-II		0.09	-	-	-	**-15.28**	-0.7	**-11.78**
Ipu-C	*162046*	-1.31	-	-	-	1.82	-1.1	**-7.25**
Ipu-I		-0.24	-	-	-	**17.64**	0.1	**6.56**
Ipu-II		0.17	-	-	-	**-16.92**	-1.32	**-13.06**
Lja-C	*150519*	-1.46	-	-	-	-0.13	-0.65	**-6.47**
Lja-I		-1.39	-	-	-	**17.98**	0.45	**9.31**
Lja-II		0.68	-	-	-	**-19.31**	-0.82	**-17.8**
Lpe-C	*135282*	-1.58	-	-	-	0.93	-1.18	**-3.02**

Lpe-I		-0.14	-	-	-	**16.49**	0.29	**9.76**
Lpe-II		0.07	-	-	-	**-14.84**	-0.58	**-12.55**
Lsa-C	*152765*	-1.8	-	-	-	0.73	-1.08	**-4.81**
Lsa-I		-0.6	-	-	-	**18.15**	-0.69	**6.45**
Lsa-II		-0.07	-	-	-	**-16**	-0.91	**-13.19**
Ltu-C	*159886*	**4.86**	-	-	-	0.06	-1.83	**-6.72**
Ltu-I		-1.21	-	-	-	**15.37**	**-5.52**	**5.84**
Ltu-II		**7.25**	-	-	-	**-14.93**	**2.98**	**-10.73**
Oni-C	*134494*	-1.58	-	-	-	0.85	-1.08	**-3.64**
Oni-I		-0.35	-	-	-	**15.98**	-0.33	**7.81**
Oni-II		-0.32	-	-	-	**-14.62**	-0.57	**-11.08**
Osa-C	*134496*	-1.58	-	-	-	1.33	-1.08	**-4.84**
Osa-I		-0.35	-	-	-	**15.92**	-0.33	**6.66**
Osa-II		-0.32	-	-	-	**-15.24**	-0.57	**-10.34**
Pvu-C	*150285*	-1.39	-	-	-	0.43	-0.7	**-7.14**
Pvu-I		-0.19	-	-	-	**19.04**	-0.65	**11**
Pvu-II		0.18	-	-	-	**-20.22**	-0.39	**-17**
Sbi-C	*140754*	-1.81	-	-	-	0.97	-1.01	**-4.53**
Sbi-I		-0.32	-	-	-	**17.14**	-0.78	**8.9**
Sbi-II		-0.31	-	-	-	**-15.1**	-0.85	**-12.66**
Shy-C	*141182*	-1.77	-	-	-	0.68	-1.12	**-3.32**
Shy-I		-0.32	-	-	-	**15.82**	1.12	**8.08**
Shy-II		-0.35	-	-	-	**-15.07**	0.68	**-11.36**
Sof-C	*141182*	-1.71	-	-	-	0.37	-1.05	**-3.89**
Sof-I		-0.53	-	-	-	**16.85**	-0.88	**8.31**
Sof-II		0.02	-	-	-	**-14.87**	-1	**-12.99**
Tae-C	*134545*	-1.57	-	-	-	0.51	-1.03	**-2.82**
Tae-I		-0.24	-	-	-	**17.71**	0.5	**9.95**
Tae-II		0.07	-	-	-	**-15.13**	-0.41	**-12.83**
Zma-C	*140384*	**-2.04**	-	-	-	0.61	-1.05	**-3.51**
Zma-I		-0.13	-	-	-	**16.93**	-0.75	**9.34**
Zma-II		-0.37	-	-	-	**-14.55**	-0.84	**-13.33**
Pho-C	*217942*	-1.06	-	-	-	**-5.98**	-1.47	**-6.17**
Pho-I		-0.67	-	-	-	**4.95**	-0.46	**5.27**
Pho-II		-1.06	-	-	-	**-12.46**	0.46	**-12.95**

Category VI

**Species**	**Len**	**$A2_C$->A1**	**A1->$A2_C$**	**A1->$A1_C$**	**$A1_C$->A1**	**$A1_C$->A2**	**A2->$A1_C$**	**$A2_C$->A2**	**A2->$A2_C$**
Cat-C	*152254*	-	-	**9.82**	**-3.64**	-	-	-	-
Cat-I		-	-	**4.96**	**6.64**	-	-	-	-
Cat-II		-	-	-1.9	**-5.89**	-	-	-	-
Chgl-C	*131183*	-	-	**3.83**	**-10.02**	-	-	-	-
Chgl-I		-	-	**22.66**	**6.18**	-	-	-	-
Chgl-II		-	-	**-20.64**	**-12.61**	-	-	-	-
Cvu-C	*184933*	-	-	**15.92**	**-2.9**	-	-	-	-
Cvu-I		-	-	**19.46**	1.57	-	-	-	-

Cvu-II		-	-	**-8.93**	**-8.33**	-	-	-	-
Mvi-C	*118360*	-	-	**22.89**	**-6.58**	-	-	-	-
Mvi-I		-	-	**21.31**	**10.39**	-	-	-	-
Mvi-II		-	-	**6.66**	**-5.66**	-	-	-	-
Ota-C	*71666*	-	-	**5.57**	-0.04	-	-	-	-
Ota-I		-	-	**8.81**	0.38	-	-	-	-
Ota-II		-	-	**-4.4**	**-9.2**	-	-	-	-
Pnu-C	*138829*	-	-	1.09	**-3.04**	-	-	-	-
Pnu-I		-	-	**18.51**	**9.76**	-	-	-	-
Pnu-II		-	-	**-20.43**	**-12.26**	-	-	-	-
Ppa-C	*122890*	-	-	**28.73**	**-4.36**	-	-	-	-
Ppa-I		-	-	**18.06**	**8.04**	-	-	-	-
Ppa-II		-	-	**2.42**	**-14.63**	-	-	-	-
Sob-C	*161452*	-	-	0.6	**-11.12**	-	-	-	-
Sob-I		-	-	**19.26**	**8.86**	-	-	-	-
Sob-II		-	-	**-5.61**	**-13.62**	-	-	-	-
Pth-C	*119707*	-0.87	-1.40	-	**-**	-	-	**-**	**-**
Pth-I		0.64	**14.71**	-	**-**	-	-	**-**	**-**
Pth-II		1.37	**-8.31**	-	**-**	-	-	**-**	**-**
Pko-C	*117190*	-1.15	-1.80	-	**-**	-	-	**-**	**-**
Pko-I		0.91	**15.37**	-	**-**	-	-	**-**	**-**
Pko-II		0.92	**-9.49**	-	**-**	-	-	**-**	**-**
Mpo-C	*121024*	-	-	**-**	-	-	**-**	**24.01**	**-6.82**
Mpo-I		-	-	**-**	-	-	**-**	**16.76**	**6.32**
Mpo-II		-	-	**-**	-	-	**-**	**-5.61**	**-10.21**
Mtr-C	*124033*	-	-	-	**-**	-0.76	**6.37**	**-**	**-**
Mtr-I		-	-	-	**-**	0.66	**10.49**	**-**	**-**
Mtr-II		-	-	-	**-**	-0.03	**4.26**	**-**	**-**

Category VII

**Species**	**Len**	**A1-A1**
Lte-C	*195081*	**4.48**
Lte-I		**7.28**
Lte-II		-0.38
She-C	*223902*	**10.3**
She-I		**13.77**
She-II		**-4.25**
Spu-C	*157089*	**-7.29**
Spu-I		-0.16
Spu-II		0.2
Zci-C	*165372*	**-6.57**
Zci-I		**-5.91**
Zci-II		**-8.51**
