# Supplementary figures and images for: A comparative approach to elucidate chloroplast genome replication
Source: BMC Genomics. 2009 May 20;10:237. doi: 10.1186/1471-2164-10-237 (PMC2695485; doi:10.1186/1471-2164-10-237)

A.

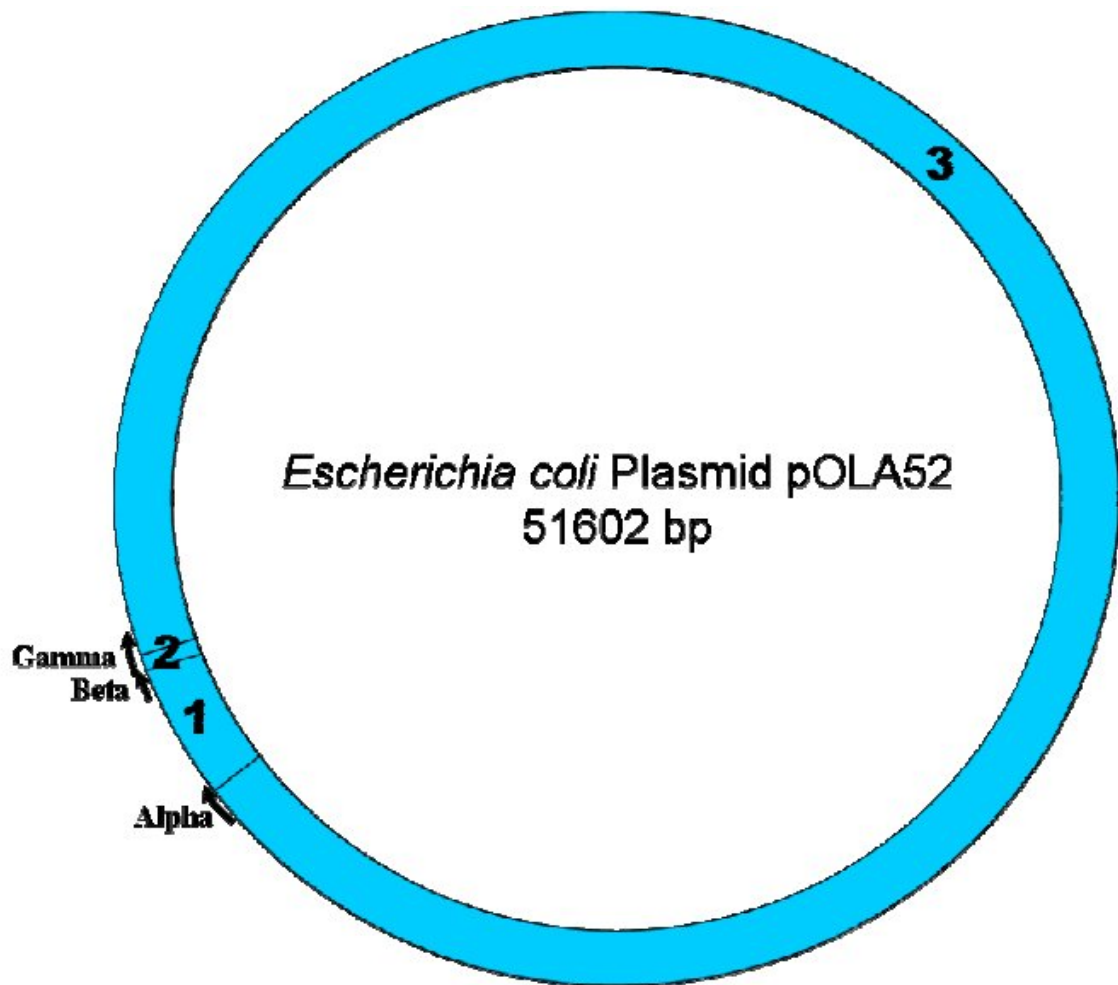

B.

| <b>Species</b>              | <b>R1</b>    | <b>R2</b>   | <b>R3</b>     |
|-----------------------------|--------------|-------------|---------------|
| <i>E. coli</i> Plasmid – C  | -1.47        | 0.27        | <b>15.45</b>  |
| <i>E. coli</i> Plasmid – I  | 1.84         | <b>-2.5</b> | <b>-11.25</b> |
| <i>E. coli Plasmid - II</i> | <b>-3.68</b> | 0.58        | <b>12.32</b>  |

Supplement: Additional File 4 — Symmetric A → G deamination gradients in E. coli plasmid. The replication origins (ori-alpha, ori-beta and ori-gamma) are mapped on the circular pOLA52 plasmid of E. coli (NC_010378) using SimVector 4.22, and the regions between these origins are annotated as R1, R2 and R3, respectively (see Additional File 4A). The t-statistics of the A → G (A/(A+G)) deamination trends are depicted in the table (see Additional File 4B, for the complete regions R1, R2 and R3 (first row for each species appended with '-C'), and after dividing these regions mid-way, for the first half (second row for each species appended with '-I') and second half (third row for each species appended with '-II') respectively for these regions. Significant values (P < 0.05, 2-tailed t-test) are emboldened. [file 1471-2164-10-237-S4.pdf]
